# The Friend of GATA Transcriptional Co-Regulator, U-Shaped, Is a Downstream Antagonist of Dorsal-Driven Prohemocyte Differentiation in *Drosophila*

**DOI:** 10.1371/journal.pone.0155372

**Published:** 2016-05-10

**Authors:** Hongjuan Gao, Rajkumar Baldeosingh, Xiaorong Wu, Nancy Fossett

**Affiliations:** 1 Center for Vascular and Inflammatory Diseases and the Department of Pathology, University of Maryland School of Medicine, Baltimore, Maryland, United States of America; 2 Graduate Program in Life Sciences, University of Maryland School of Medicine, Baltimore, Maryland, United States of America; National Cancer Institute, UNITED STATES

## Abstract

Recent studies suggest that mammalian hematopoietic stem and progenitor cells (HSPCs) respond directly to infection and inflammatory signaling. These signaling pathways also regulate HSPCs during steady-state conditions (absence of infection), and dysregulation may lead to cancer or age-related loss of progenitor repopulation capacity. Toll-like receptors (TLRs) are a major class of pathogen recognition receptors, and are expressed on the surface of immune effector cells and HSPCs. TLR/NF-κB activation promotes HSPCs differentiation; however, the mechanisms by which this signaling pathway alters the intrinsic transcriptional landscape are not well understood. Although *Drosophila* prohemocytes are the functional equivalent of mammalian HSPCs, a prohemocyte-specific function for Toll signaling has not been reported. Using *Drosophila* transgenics, we identified prohemocyte-specific roles for Toll pathway members, Dorsal and Cactus. We showed that Dorsal is required to limit the size of the progenitor pool. Additionally, we showed that activation of Toll signaling in prohemocytes drives differentiation in a manner that is analogous to TLR/NF-κB-driven HSPC differentiation. This was accomplished by showing that over-expression of Dorsal, or knockdown of Cactus, promotes differentiation. We also investigated whether Dorsal and Cactus control prohemocyte differentiation by regulating a key intrinsic prohemocyte factor, U-shaped (Ush), which is known to promote multipotency and block differentiation. We showed that Dorsal repressed Ush expression levels to promote differentiation, whereas Cactus maintained Ush levels to block differentiation. Additionally, we showed that another Toll antagonist, Lesswright, also maintained the level of Ush to block differentiation and promote proliferative quiescence. Collectively, these results identify a novel role for Ush as a downstream target of Toll signaling.

## Introduction

A growing body of evidence suggests that mammalian hematopoietic stem and progenitor cells (HSPCs) respond directly to infection and inflammatory signaling [1]. Toll-like receptors (TLRs), a major class of pathogen recognition receptors, are expressed on the surface of immune effector cells and HSPCs [2–5]. Furthermore, activation of this signaling pathway drives HSPCs toward myeloid differentiation [5]. Nevertheless, the mechanisms by which TLR/NF-κB signaling alters the intrinsic transcriptional landscape to promote HSPC differentiation are not well understood. In addition, it is not known if the TLR/NF-κB signaling cascade regulates HSPCs during steady-state hematopoiesis. To begin to address these questions, we used the *Drosophila* hematopoietic system to better understand TLR/NF-κB regulation of hematopoietic progenitors. *Drosophila* hematopoietic progenitors (prohemocytes) share key characteristics with mammalian HSPCs, including multipotency, quiescence and niche-responsiveness [6–8]. Furthermore, many of the regulatory strategies that control HSPC differentiation are conserved [9]. However, unlike HSPCs, a prohemocyte function for Toll signaling has not been reported.

Similar to vertebrate hematopoiesis, *Drosophila* hematopoiesis takes place in multiple tissues, including hub cells within the adult abdomen, the embryonic head mesoderm, the larval body wall, and a specialized organ known as the larval lymph gland [6,10–15]. The lymph gland contains a pair of primary lobes and a series of secondary lobes. The primary lobe is organized into the following three well defined functional regions or zones: the Posterior Signaling Center (PSC), the medullary zone (MZ), and the cortical zone (CZ). The PSC resides at the base of the primary lobe and relays signals that regulate prohemocytes and their descendant lineages [7,8,16,17]. Prohemocytes are located within the MZ, and the descendant lineages are generally restricted to the CZ (S1 Fig). This zonal arrangement enables analyses to be conducted with prohemocytes that retain their interaction with the micro-environment, including the PSC and descendant lineages. This spatial arrangement also facilitates the study of communication between different cell types and key signaling pathways in the context of the whole organism [6–8,16,18].

Prohemocytes give rise to three classes of terminally differentiated blood cell types known as plasmatocytes, crystal cells, and lamellocytes. Plasmatocytes are operational macrophages and constitute the major portion of the cellular blood component. Crystal cells are named for their crystalline inclusion bodies. These cells secrete antimicrobials and function during wound healing [10,19]. Stress conditions, such as immune challenge, promote the differentiation of lamellocytes. However, lamellocytes are rarely observed under steady-state conditions [19–21].

In *Drosophila*, Toll signaling can promote lamellocyte differentiation in response to infection. Moreover, mutations that constitutively activate the Toll pathway significantly increase lamellocyte differentiation [22–29]. Toll is a transmembrane receptor that is part of a signaling cascade required for immunity as well as for establishing dorsal-ventral polarity in the *Drosophila* embryo [22–24,26,28,30–37]. The signaling cascade is activated when Toll binds the ligand, Spätzle. The activated Toll receptor then binds to the cytoplasmic adaptor protein, MyD88. MyD88 recruits Tube and Pelle to form a complex that functions to promote phosphorylation and degradation of Cactus (Cact). Cact sequesters the transcription factors Dorsal and Dorsal-related immunity factor (Dif) in the cytoplasm. Degradation of Cact releases these factors, permitting translocation to the nucleus and regulation of inflammation-associated target genes [33,34,38–42]. Dorsal and Dif have overlapping functions in cellular immunity and steady-state hematopoiesis [22,29,43–45]. During steady-state hematopoiesis, the loss of both Dorsal and Dif dramatically reduces hemocyte proliferation and survival [43,45]; however, loss of both factors has been shown to increase crystal cell number [22].

Striking similarities exist between *Drosophila* Toll signaling and mammalian TLR signaling. As seen with Toll signaling, TLR activation leads to degradation of IκB (Cact), which permits translocation of NF-κB (Dorsal, Dif) to the nucleus and regulation of genes that mediate inflammation and cellular immunity. Moreover, core downstream effectors of the Toll signaling cascade are conserved, including MyD88, IRAK-1 (Pelle) and IRAK-4 (Tube) [46,47].

In this report, we used *Drosophila* to advance our understanding of how TLR/NF-κB signaling regulates HSPCs. We first identified prohemocyte-specific roles for Toll pathway members Dorsal and Cact. Importantly, we showed that Dorsal is required to limit the size of the prohemocyte pool during steady-state conditions. This finding supports the idea that members of inflammatory signaling pathways regulate hematopoietic progenitors during steady-state conditions. However, we observed that Dif is not required to limit the size of the prohemocyte pool. We showed that over-expression of Dorsal or knockdown of Cact in prohemocytes reduced the size of the MZ and increased lamellocyte differentiation. These results demonstrated that TLR/NF-κB-driven HSPC differentiation is conserved, which provided the rationale for using *Drosophila* to identify factors that interface with NF-κB to regulate HSPCs. U-shaped (Ush) is a Friend of GATA (FOG) transcriptional co-regulator that binds the GATA factor Serpent (Srp) to form a complex that limits prohemocyte differentiation [48–51]. Loss of Ush leads to a dramatic reduction in the prohemocyte pool and increased lamellocyte differentiation [50,52]. This phenotype is strikingly similar to the activated Dorsal phenotype, and suggested that Dorsal promotes prohemocyte differentiation by inhibiting Ush. We showed that Dorsal represses Ush expression levels, whereas Cact is required to maintain Ush levels. Moreover, Ush antagonizes the ability of Dorsal to drive prohemocyte differentiation, and Ush acts with Cact to limit differentiation. Finally, we showed that the Toll antagonist, Lesswright (Lwr; Ubc9) [45,53], is required to maintain Ush levels and that Ush and Lwr interact to block lamellocyte differentiation and promote proliferative quiescence. These findings support the idea that Toll signaling controls the level of Ush as a means to regulate prohemocyte choice between multipotency and differentiation. During vertebrate hematopoiesis, the GATA-1:FOG-1 complex limits myeloid lineage differentiation, including eosinophils, mast cells, and granulocytes [54–56]. Thus, FOG antagonism of TLR signaling may be an important conserved strategy that limits the inflammatory response. We have identified an important connection between Toll signaling and the intrinsic progenitor regulatory machinery that will provide a framework to further investigate how HSPCs are regulated by inflammatory pathways during steady state hematopoiesis.

## Materials and Methods

### Fly strains

The *w*^*1118*^ strain served as the wild-type stock for these studies. The following strains were generous gifts from colleagues: *domeless-Gal4* from M. Crozatier (University Paul Sabatier); *Tep4-Gal4*, *Eater-Gal4* and *MSNF9mo-DsRed* from T. Tokusumi and R. A. Schulz (University of Notre Dame); *Antp-Gal4*, *yw; cn Dif*^*1*^ *bw* and *yw; cn Dif*^*2*^ *bw/ CyO* from S. Govind (City College of New York). The following strains were obtained from the Bloomington Stock Center: ^*dl1 cn1 sca1/CyO*, *l(2)DTS1001*^, ^y1 w*; P{w[+mC]=UAS-dl.*H}2*^, *y*^*1*^ sc* *v*^*1*^*; P{TRiP*.*HMS00084}attP2* (*UAS-cact*^*RNAi*^), *y*^*1*^ sc* *v*^*1*^*; P {TRiP*.*HMS00028}attP2* (*UAS-dl*^*RNAi*^), *y*^*1*^ sc* *v*^*1*^*; P {TRiP*.*HMS00727}attP2* (*UAS-dl*^*RNAi*^), *y*^*1*^ *v*^*1*^; *P {TRiP*.*HM05191}attP2* (*UAS-Dif*^*RNAi*^), *P {TRiP*.*HM05257}attP2* (*UAS-Dif*^*RNAi*^), ^*cact1 cn1 bw1/CyO*, *l(2)DTS5131*^. ^y1 w*; lwr5 b1 cn1 bw1/CyO, *y+*^,*y1* sc* *v1*; *P{TRiP*.*HMS01648}attP40*, (*UAS-lwr*^*RNAi*^). The following stocks have been previously described: *y w*^*67c23*^*; ush*^*vx22*^*/CyO y*^*+*^, *y w*^*67c23*^; *ush*^*R24*^*/CyO y*^*+*^, *y w; UAS-ush*, *UAS- ush*^*RNAi*^ [50,51]. *w; UAS-ush; UAS-cact*^*RNAi*^ males were produced using the following strategy: First, *w; UAS-ush; TM3*, *Sb/TM6*, *Tb* and *w; Sco/CyO; UAS-cact*^*RNAi*^ males and females were produced and mated. Second, male and virgin female progeny with the w; *UAS-ush*/CyO; *UAS-cact*^*RNAi*^ */TM3*, *Sb* genotype were selected and mated. Third, male progeny with the *w; UAS-ush; UAS-cact*^*RNAi*^ genotype were selected and used in these studies.

### Gene expression analyses

Gene expression analyses were conducted using lymph glands from mid-third instar larvae (collected 96 to 104 hours after egg laying). As indicated in specific experiments, gene expression analyses were also conducted using lymph glands from either early-third instar larvae (collected 78 to 86 hours after egg laying) or late-third instar larvae (collected 112 to 120 hours after egg laying). All control and experimental samples were age-matched and cultured on standard media at 23°C. The UAS/Gal4 binary system [57] was used to express transgenes in a tissue-specific manner. Controls for these experiments included the Gal4 drivers crossed to *w*^*1118*^ mates. We have observed that the *dome-Gal4* and *Tep-Gal4* drivers can be used interchangeably [51]. However, the *Tep-Gal4* driver was used in experiments designed to over-express *dl* or *ush* because *dome-Gal4* driven *UAS-dl* or *UAS-ush* die during the early larval stages.

### Immunofluorescence

The dissection and fixation of larval lymph glands were performed as previously described [50]. Rabbit anti-Odd was a generous gift from J. Skeath (Washington University School of Medicine, [58] and used at a 1:4,000 dilution. Mouse anti-Attila (L1) [59] was a generous gift from I. Ando (Biological Research Center of the Hungarian Academy of Sciences) and used at a 1:50 dilution. Rabbit anti-prophenoloxidase A1 (anti-ProPO) was a generous gift from F. C. Kafatos (EMBL, [60] and used at a 1:100 dilution. Rabbit anti-U-shaped was used at a 1:4,000 dilution [48]. Rabbit anti-Serpent was used at a 1:8,000 dilution [51]. Rabbit anti-phosphohistone H3 was obtained from Cell Signaling and used at a concentration of 1:100. Mouse anti-β-galactosidase was obtained from Promega and used at a concentration of 1:2000. Mouse anti-Dorsal, anti-Cactus and anti-Wingless were obtained from the Developmental Studies Hybridoma Bank and used at a concentration of 10 μg/ml. Alexafluor-555, -568, or -488-conjugated secondary antibodies directed against rabbit or mouse (Invitrogen) were used at a 1:2,000 dilution. Fluorescence was captured, analyzed and recorded using Olympus confocal microscopy or Zeiss Axioplan optics. The relative expression was determined from the densitometric mean values calculated for fluorescent antibody staining using Zeiss Axiovision software as previously described [50]. The size of the medullary zone was determined by measuring the area of Odd-expressing cells. The number and percentage of crystal cells and HP3-expressing cells were analyzed using Zeiss Axioplan software as previously described [61]. The statistical significance was evaluated using the Student’s t-test or one way Anova. At least 20 primary lymph gland lobes were sampled, and each assay consisted of at least 10 control and 10 experimental samples. In our hands, control lymph glands have an average of 1 lamellocyte per lymph gland lobe. However, lamellocytes can form large aggregates making it difficult to obtain accurate cell counts. For this reason, we scored primary lymph gland lobes positive for aberrant lamellocyte differentiation when aggregates were greater than 300 μm^2^ or more than 5 individual lamellocytes were visible [61]. Again, at least 20 primary lymph gland lobes were sampled, and each assay consisted of at least 10 control and 10 experimental samples. When comparing 2 samples, statistical significance was evaluated using aberrant differentiation as a categorical variable for experimental and control samples in 2x2 contingency tables and P values were calculated using Fisher’s Exact test. When comparing 3 samples, 2x3 contingency tables were used and P values were calculated using Chi-square test. The number of Odd-expressing cells was determined by counting the number of Odd-positive nuclei from 6 to 8 control and 6 to 8 experimental lymph glands (12 to 16 total). The statistical significance was evaluated using the Student’s t-test. All of the measurements and statistical analyses are deposited in S1 File.

## Results

### Toll pathway members Dorsal and Cact function in prohemocytes to regulate the choice between multipotency and differentiation

**Fig 1:**
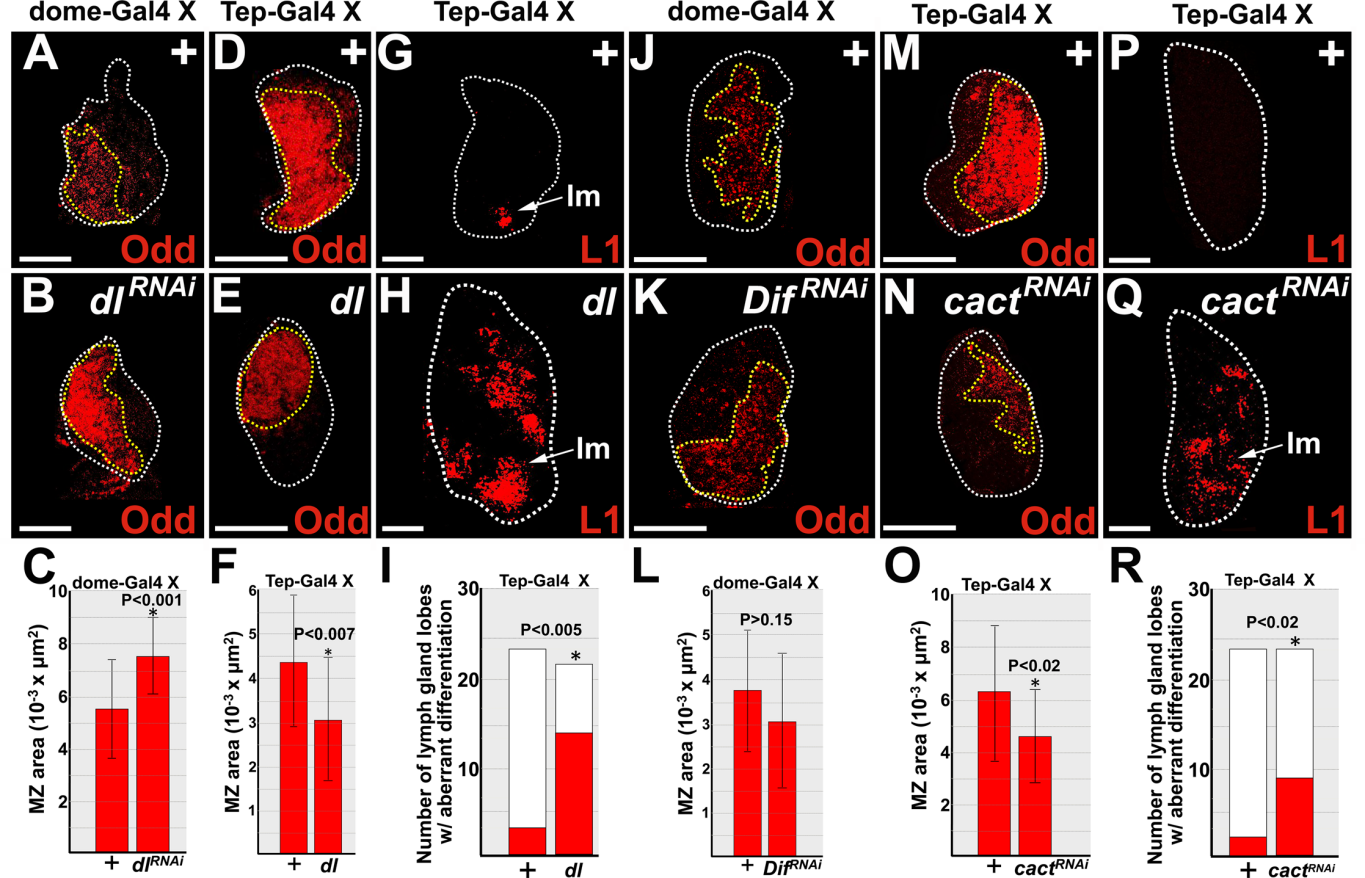
Dorsal and Cact regulate prohemocyte differentiation. **(A-C,J-L)** *dome-Gal4* or (**D-I,M-R**) *Tep-Gal4* females were crossed to control (+) males or males that carry *UAS*-*dl*, *UAS*-*dl*^*RNAi*^, *UAS-Dif*^*RNAi*^, or *UAS-cact*^*RNAi*^ transgenes. (**A,B**) Knockdown of Dorsal (*dl*^*RNAi*^) significantly increased the size of the MZ compared to controls (+). (**C**) Histogram showing the size of the MZ significantly increased in lymph glands in which Dorsal was knocked down (*dl*^*RNAi*^). Student’s t-test; error bars show standard deviation; P values are as shown; control and *dl*^*RNAi*^ (n = 18). (**D,E**) In contrast, over-expression of Dorsal (*dl*) significantly reduced the size of the MZ compared to controls (+). (**F**) Histogram showing the size of the MZ was significantly reduced in lymph glands in which Dorsal was over-expressed. Student’s t-test; error bars show standard deviation; P values are as shown; control and *dl* (n = 21). (**G,H**) Over-expression of Dorsal significantly increased lamellocyte differentiation. (**I**) Histogram showing that aberrant lamellocyte differentiation was significantly increased when Dorsal was over-expressed. Fisher’s Exact test; P value is as shown; control (n = 24), *dl* (n = 22). (**J,K**) Knockdown of Dif (*Dif*^*RNAi*^*)* had no effect on the size of the MZ. (**L**) Histogram showing the size of the MZ was not affected in lymph glands in which Dif was knocked down. Student’s t-test; error bars show standard deviation; control and *Dif*^*RNAi*^ (n = 19); P values are as shown. (**M,N**) Knockdown of Cact (*cact*^*RNAi*^) significantly reduced the size of the MZ compared to controls (+). (**O**) Histogram showing the size of the MZ was significantly reduced in lymph glands in which Cact was knocked down. Student’s t-test; error bars show standard deviation; P values are as shown; control and *cact*^*RNAi*^ (n = 24). (**P,Q**) Knockdown of Cact significantly increased lamellocyte differentiation. (**R**) Histogram showing that the number of primary lymph gland lobes with aberrant lamellocyte differentiation was significantly greater when Cact was knocked down. Fisher’s Exact test; P value is as shown; control and *cact*^*RNAi*^ (n = 24). Arrows mark lamellocytes (lm). White dotted lines delineate the entire lymph gland; yellow dotted lines delineate the prohemocyte pool. Scale bars: 50 μm. (**A-F, J-O**) mid-third instar larvae; (**G-I, P-R**) late-third instar larvae.

Activated TLR/NF-κB signaling promotes mammalian HSPC differentiation [5]. In *Drosophila*, the cognate Toll signaling pathway regulates both the response to infection and aspects of steady-state hematopoiesis [22,23,26,28,35–37]. The lymph gland MZ prohemocytes are the functional equivalent of mammalian HSPCs [62]; however, unlike HSPCs, a prohemocyte-specific function for Toll signaling has not been reported. Furthermore, Toll pathway members Dorsal and Spätzle are expressed throughout the lymph gland [22,63]. Therefore, we tested if Dorsal acts in prohemocytes to regulate differentiation. The level of Dorsal expression was specifically altered in prohemocytes using the UAS/Gal4 system [57]. MZ prohemocytes were monitored using the specific marker, Odd [61]. First, we confirmed that Dorsal was indeed knocked down by showing that the level of expression was significantly reduced in *dome-Gal4* driven *UAS-dl*^*RNAi*^ lymph glands compared to controls (S2 Fig). We then showed that knockdown of Dorsal in prohemocytes produced a statistically significant increase the size of the MZ (Fig 1A–1C). In contrast, over-expression of Dorsal produced a statistically significant decrease in the size of the prohemocyte pool and an increase in lamellocyte differentiation (Fig 1D–1I). These results suggest that during steady-state conditions, Dorsal functions in prohemocytes to limit population size by promoting differentiation.

Given that Dorsal and Dif have overlapping functions during both the immune response and steady-state hematopoiesis [29,43–45], we tested if Dif was also required to limit the size of the prohemocyte pool. We used the UAS/Gal4 system to knockdown Dif in prohemocytes, and observed that there was no significant change in the size of the MZ (Fig 1J–1L). We confirmed this result by showing that expression of an alternate *UAS-Dif*^*RNAi*^ allele did not change the size of the MZ. We also observed that MZ size did not change in *Dif* heterozygotes (S2 Fig). Collectively, these results show that Dif does not regulate the size of the MZ and that Dorsal acts independently of Dif to control the size of the MZ.

Cact is known to antagonize the function of Dorsal and loss of Cact activates Dorsal [33,34]. Thus, to confirm that activation of Dorsal drives prohemocyte differentiation, we knocked down Cact in prohemocytes using the UAS/Gal4 system combined with RNAi technology. First, we confirmed that Cact was indeed knocked down by showing that the level of expression was significantly reduced in *Tep-Gal4* driven *UAS-cact*^*RNAi*^ lymph glands compared to controls (S2 Fig). We then showed that knockdown of Cact produced a statistically significant decrease in the size of the MZ and an increase in lamellocyte differentiation (Fig 1M–1R). In addition, we showed that *dome-Gal4* driven *UAS-cact*^*RNAi*^ also increased lamellocyte differentiation (S2 Fig). Thus, Cact is required during steady-state conditions to block lamellocyte differentiation and maintain the size of the MZ. Manipulating the expression of Dorsal and Cact could have altered the size of the MZ by affecting prohemocyte cell size and not cell number. However, we think this is unlikely. We sampled at least 6 control and 6 experimental lymph glands from each experiment and observed that the increase in MZ size corresponded to a statistically significant increase in cell number, whereas a decrease in MZ size corresponded to a statistically significant decrease in cell number (S2 Fig). Thus, the observed change in MZ size most likely reflects a change in the number of the prohemocytes rather than a change in cell size. Collectively, these results show that Dorsal and Cact have opposing functions in the control of prohemocyte differentiation and confirm that Dorsal promotes prohemocyte differentiation (S2 Fig).

### Dorsal and Cact regulate Ush protein levels to control prohemocyte differentiation

**Fig 2:**
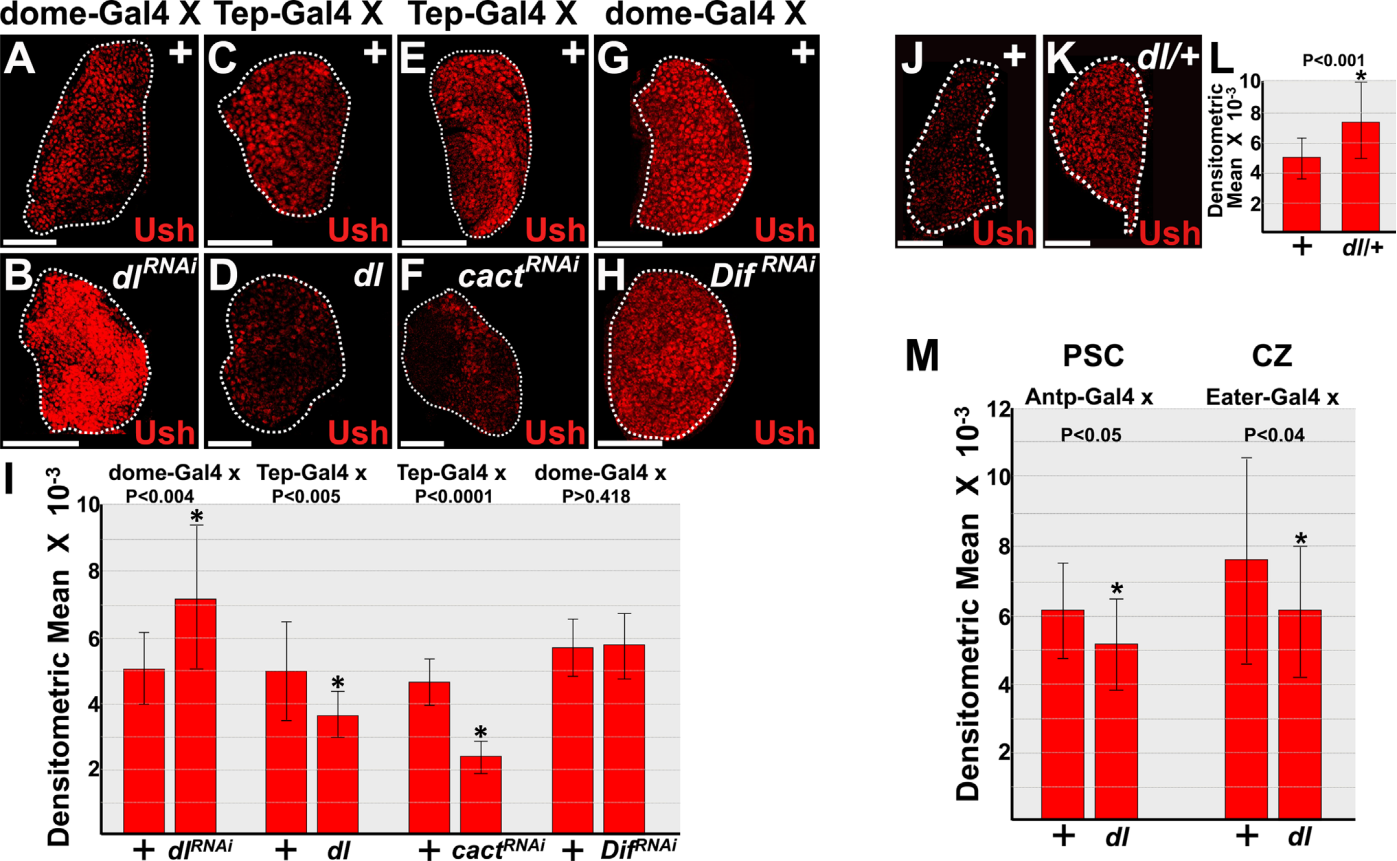
Dorsal controls the level of Ush protein expression. (**A-I**) Prohemocyte-specific expression of *UAS*-*dl*^*RNAi*^, *UAS*-*dl*, *UAS-cact*^*RNAi*^, or *UAS-Dif*^*RNAi*^ transgenes. (**A,B,G,H**) *dome-Gal4* or (**C-F**) *Tep-Gal4* females were crossed to control (+) males or males that carry *UAS*-*dl*^*RNAi*^, *UAS*-*dl*, *UAS-cact*^*RNAi*^, or *UAS-Dif*^*RNAi*^ transgenes. (**A,B**) Knockdown of Dorsal (*dl*^*RNAi*^) in prohemocytes significantly increased Ush levels compared to controls (+). (**C,D**) In contrast, over-expression of Dorsal (*dl*) in prohemocytes significantly reduced Ush levels compared to controls (+). (**E,F**) Likewise, knockdown of Cact (*cact*^*RNAi*^) in prohemocytes significantly reduced Ush levels compared to controls. (**G,H**) However, knockdown of Dif (*Dif*^*RNAi*^) in prohemocytes did not alter the level of Ush compared to controls. (**I**) Histogram showing the relative level of Ush expression in lymph glands with altered expression of Toll pathway members. Control and *dl*^*RNAi*^ (n = 14); control and *dl* (n = 15); control and *cact*^*RNAi*^ (n = 17); control and *Dif*^*RNAi*^ (n = 15). Student’s t-test; error bars show standard deviation; P values are as shown. (**J-M**) Dorsal regulation of Ush expression varies across hematopoietic compartments. **(J,K)** Ush expression increased in (**K**) *dl* heterozygous (*dl/+*) lymph glands compared to (**J**) controls (+). (**L**) Histogram showing the level of Ush expression is significantly increased in *dl/+* compared to controls. Student’s t-test; error bars show standard deviation; P value is as shown; control and *dl*/+ (n = 19). (**M**) Histogram showing the relative level of Ush expression in lymph glands in which *dl* was over-expressed in either the posterior signaling center (PSC) or cortical zone (CZ). Control and *Antp*>*dl* (n = 15); control and Eater>*dl* (n = 19). Student’s t-test; error bars show standard deviation; P values are as shown. White dotted lines delineate the entire lymph gland. Scale bars: (**A,B,E-H,J,K**) 50 μm; (**C,D**) 25 μm. Lymph glands dissected from mid-third instar larvae.

We next investigated how Dorsal and Cact alter the intrinsic regulatory landscape to control prohemocyte differentiation and population size. Ush is an intrinsic regulator that is required to maintain the prohemocyte pool and block differentiation [50]. Loss of Ush leads to a dramatic reduction in the prohemocyte population and increased lamellocyte differentiation [50,52]. This phenotype is strikingly similar to the activation of Dorsal or loss of Cact phenotypes. These observations suggested that Dorsal promotes prohemocyte differentiation by inhibiting Ush. To address this question, we first determined if Dorsal downregulates Ush expression. Knockdown of Dorsal in the MZ increased the level of Ush, whereas over-expression of Dorsal decreased Ush levels (Fig 2A–2D and 2I). In addition, knockdown of Cact in the MZ decreased the level of Ush (Fig 2E and 2F and 2I). Thus, Cact antagonizes the function of Dorsal to control the level of Ush. On the other hand, knockdown of Dif in the MZ did not affect the level of Ush (Fig 2G–2I). Thus, Dorsal functions independently of Dif to limit both Ush expression and the size of the MZ.

Dorsal is expressed throughout the lymph gland [22]. We observed that Ush levels in the lymph gland increased in *dl* heterozygotes (Fig 2J–2L). A previous study showed that Dorsal acts in the PSC to promote lamellocyte production in response to parasitic wasp infection [22]. Collectively, these observations raised the possibility that in addition to prohemocytes, Dorsal may also function in the PSC and/or differentiating CZ cells to repress Ush protein expression levels. To test this hypothesis, we used the UAS/Gal4 system to over-express Dorsal in either the PSC or CZ and observed a statistically significant decrease in the level of Ush (Fig 2M).

We previously identified cis-regulatory modules (CRMs) that regulate *ush* gene expression and contain consensus Dorsal binding sites [64] (data not shown). This suggested that Dorsal downregulates Ush by directly repressing gene expression. As shown in Fig 2J–2L, Ush protein expression significantly increased in *dl* heterozygotes. Thus, if Dorsal acts to repress *ush* gene expression by binding to the *ush CRM*, then *CRM lacZ* transgene activity should also increase in *dl* heterozygotes. We tested five different *ush CRM-reporter lacZ* transgenes, including the 7.5 kb CRM that recapitulates *ush* gene expression in most embryonic tissues and throughout the lymph gland [64]. None of the transgenes exhibited increased activity in *dl* heterozygotes (S3 Fig and data not shown). These results suggest that Dorsal does not repress *ush* gene expression through consensus binding sites within the CRM.

**Fig 3:**
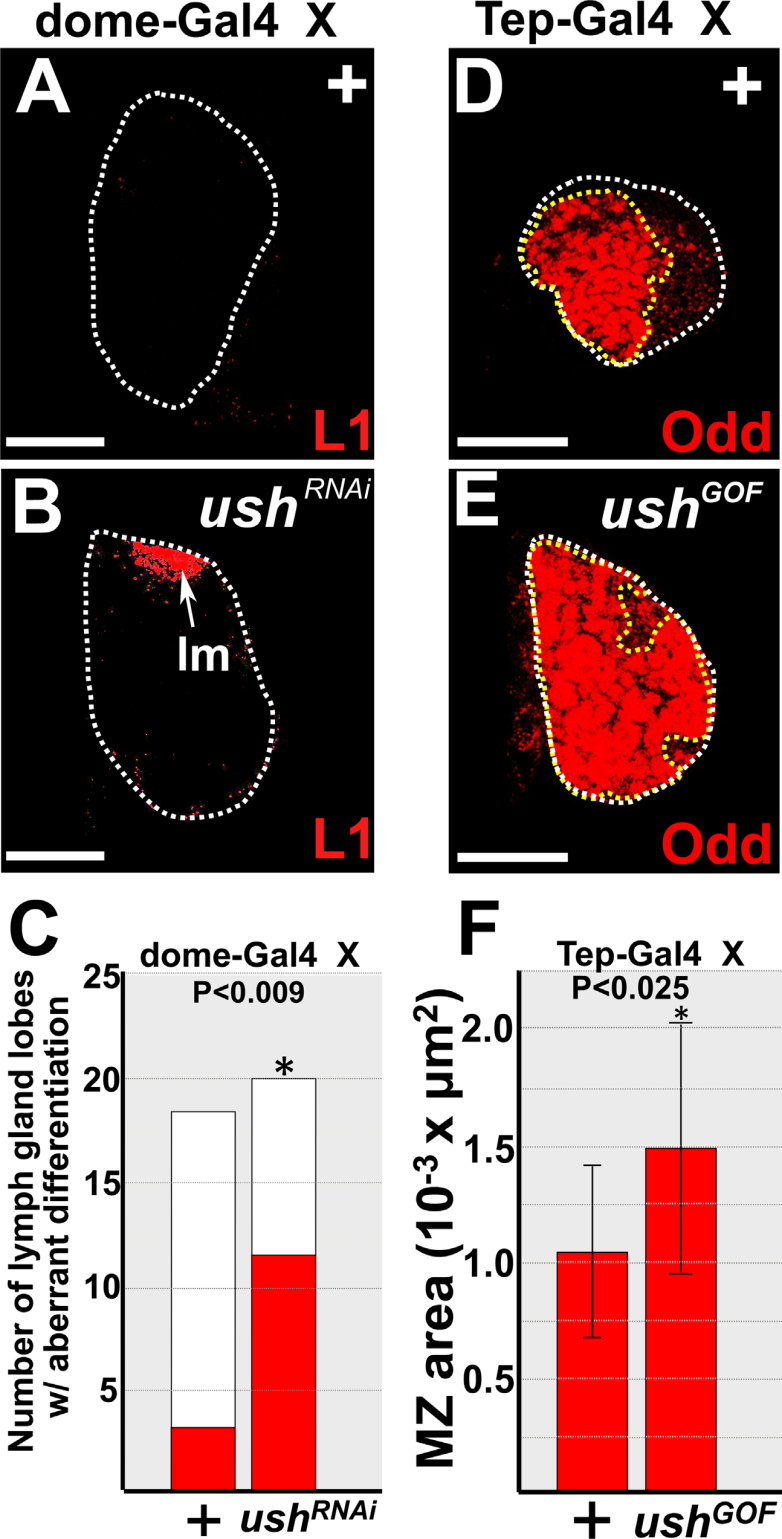
Ush functions in prohemocytes to promote multipotency and block differentiation. (**A-C**) Knockdown of Ush (*ush*^*RNAi*^) significantly increased lamellocyte differentiation compared to controls (+). *dome-Gal4* females were crossed to control (+) males or males that carry *UAS*-*ush*^*RNAi*^ transgene. Arrow marks lamellocytes (lm). (**C**) Histogram showing that lamellocyte differentiation was significantly increased in *dome-Gal4* driven *UAS-ush*^*RNAi*^ lymph glands compared to controls. Fisher’s Exact test; P value is as shown; controls (n = 18); *ush*^*RNAi*^ (n = 20). (**D-F**) Over-expression of Ush in prohemocytes significantly increased the size of the MZ. *Tep-Gal4* females were crossed to control (+) males or males that carry the *UAS*-*ush* transgene. (**F**) Histogram showing the size of the MZ significantly increased in lymph glands in which Ush was over-expressed (*ush*^*GOF*^). Student’s t-test; error bars show standard deviation; P values are as shown; control and *ush*^*GOF*^ (n = 11). White dotted lines delineate the entire lymph gland; yellow dotted lines delineate the MZ. Scale bars: (**A,B**) 100 μm; (**D,E**) 25 μm. (**A-C**) Lymph glands from late-third instar larvae; (**D-F**) lymph glands from mid-third instar larvae.

Ush is expressed throughout the lymph gland. To demonstrate that Ush functions in prohemocytes to regulate the choice between multipotency and differentiation, we altered the level of Ush in the MZ using the UAS/Gal4 binary system [57]. Knockdown of Ush increased lamellocyte differentiation (Fig 3A–3C) [51], whereas overexpression of Ush increased the size of the MZ (Fig 3D–3F). Again, we sampled 6 control and 6 experimental lymph glands and observed that prohemocyte number was significantly increased in Ush gain-of-function lymph glands compared to controls (S4 Fig). This strongly suggests that increased MZ size resulted from an increase in number of prohemocytes rather than increased in prohemocyte cell size. Thus, Ush functions in the MZ to block differentiation and maintain the prohemocyte pool.

**Fig 4:**
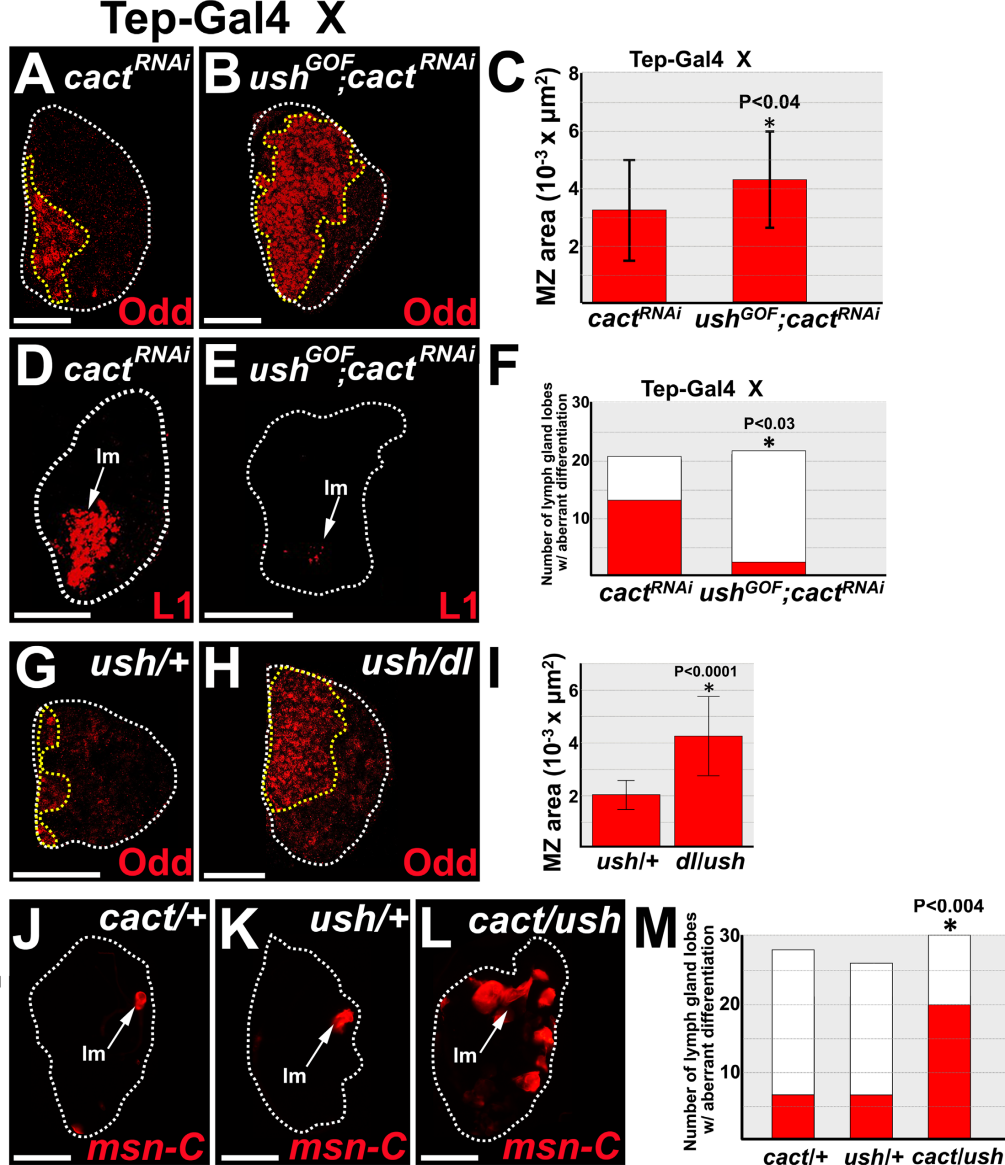
Ush acts with Cact to limit Dorsal-driven prohemocyte loss and differentiation. (**A-F**) Over-expression of Ush rescues Cact knockdown-induced prohemocyte loss and lamellocyte differentiation. *Tep-Gal4* females were crossed to males that carry (**A,D**) the *UAS-cact*^*RNAi*^ (*cact*^*RNAi*^) transgene or (**B,E**) both the *UAS-ush* (*ush*^*GOF*^) and *UAS-cact*^*RNAi*^ transgenes. (**B**) Co-expression of *ush* and *cact*^*RNAi*^ transgenes significantly increased the size of the MZ compared to (**A**) expression of the *cact*^*RNAi*^ transgene alone. (**C**) Histogram showing the size of the MZ significantly increased in lymph glands with *Tep-Gal4* driven co-expression of *UAS*-*ush* and *UAS-cact*^*RNAi*^ compared to *Tep-Gal4* driven expression of *UAS*-*cact*^*RNAi*^ alone. Student’s t-test; error bars show standard deviation; P values are as shown; *ush* with *cact*^*RNAi*^ and *cact*^*RNAi*^ alone (n = 22). (**E**) Co-expression of *ush* and *cact*^*RNAi*^ transgenes significantly reduced lamellocyte differentiation compared to (**D**) expression of the *cact*^*RNAi*^ transgene alone. (**F**) Histogram showing the number of lymph gland lobes with aberrant lamellocyte differentiation was significantly greater in lymph glands with *Tep-Gal4* driven *UAS*-*cact*^*RNAi*^ compared to *Tep-Gal4* driven co-expression of *UAS*-*ush* and *UAS-cact*^*RNAi*^. Fisher’s exact test; P values are as shown; *cact*^*RNAi*^ alone (n = 21), *ush* with *cact*^*RNAi*^ (n = 22). (**G-I**) The size of the MZ was significantly larger in (**H**) *dl/ush* trans-heterozygous lymph glands compared to (**G**) *ush/+* lymph glands. (**I**) Histogram showing the size of the MZ in *ush/+* and *dl/ush* lymph glands. Student’s t-test; error bars show standard deviation; P values are as shown; *ush/+* and *dl/ush* (n = 18). **(J-M)** Ush and Cactus act together to block lamellocyte differentiation. (**L**) Lamellocyte differentiation in *cact/ush* trans-heterozygous lymph glands was significantly increased compared to either (**J**) *cact/+* or (**K**) *ush/+* lymph glands. (**M**) Histogram showing that the number of primary lymph gland lobes with aberrant lamellocyte differentiation was significantly greater in *cact/ush* trans-heterozygous (n = 30) lymph glands compared to either *cact/+* (n = 28) or *ush/+* (n = 26) lymph glands. Chi-square test; P value is as shown. White dotted lines delineate the entire lymph gland; yellow dotted lines delineate the prohemocyte pool. Arrows mark lamellocytes (lm). (**A-C,G-I**) Mid-third instar larvae; **(D-F,J-M**) late-third instar larvae. Scale bars: (**A-C,G-I**) 50 μm; (**D-F,J-M**) 100 μm.

We next asked if Dorsal represses Ush in order to promote prohemocyte differentiation. If this is the case, then over-expression of Ush should reverse Dorsal-driven prohemocyte loss and differentiation. To test this hypothesis, we used *Tep-Gal4* to co-express wild-type *ush* and *cact*^*RNAi*^ or *cact*^*RNAi*^ alone in prohemocytes and assessed prohemocyte differentiation for each genotype. Co-expression of *ush* with *cact*^*RNAi*^ increased MZ size and limited lamellocyte differentiation compared to expression of *cact*^*RNAi*^ alone (Fig 4A–4F). Thus, over-expressing Ush in prohemocytes counteracts Dorsal-driven prohemocyte differentiation. We then tested if lowering the level of Dorsal rescues *ush* mutant-induced prohemocyte loss. The size of the MZ was significantly increased in the lymph glands of *dl/ush* trans-heterozygotes compared to *ush* heterozygotes (Fig 4G–4I). Furthermore, lymph glands with increased MZ size (Fig 4A–4C and 4G–4I) also had increased numbers of prohemocytes (S4 Fig). This strongly suggested that the change in MZ size was due to change in prohemocyte number and not prohemocyte cell size. Together, these data provide strong support for the hypothesis that Ush antagonizes Dorsal-driven prohemocyte loss and differentiation. Given that Cact also antagonizes Dorsal, lowering the dose of both Ush and Cact should increase Dorsal function, leading to increased lamellocyte differentiation. To test this hypothesis, we assessed lamellocyte differentiation in *cact/ush* trans-heterozygotes. We observed a significant increase in lamellocyte differentiation in trans-heterozygotes compared to either *cact* or *ush* heterozygotes (Fig 4J–4M). These results, coupled with our observation that Dorsal represses Ush protein levels, strongly suggest that Ush is a downstream antagonist of Dorsal function and that Dorsal and Cact control prohemocyte differentiation by regulating the level of Ush (S4 Fig).

**Fig 5:**
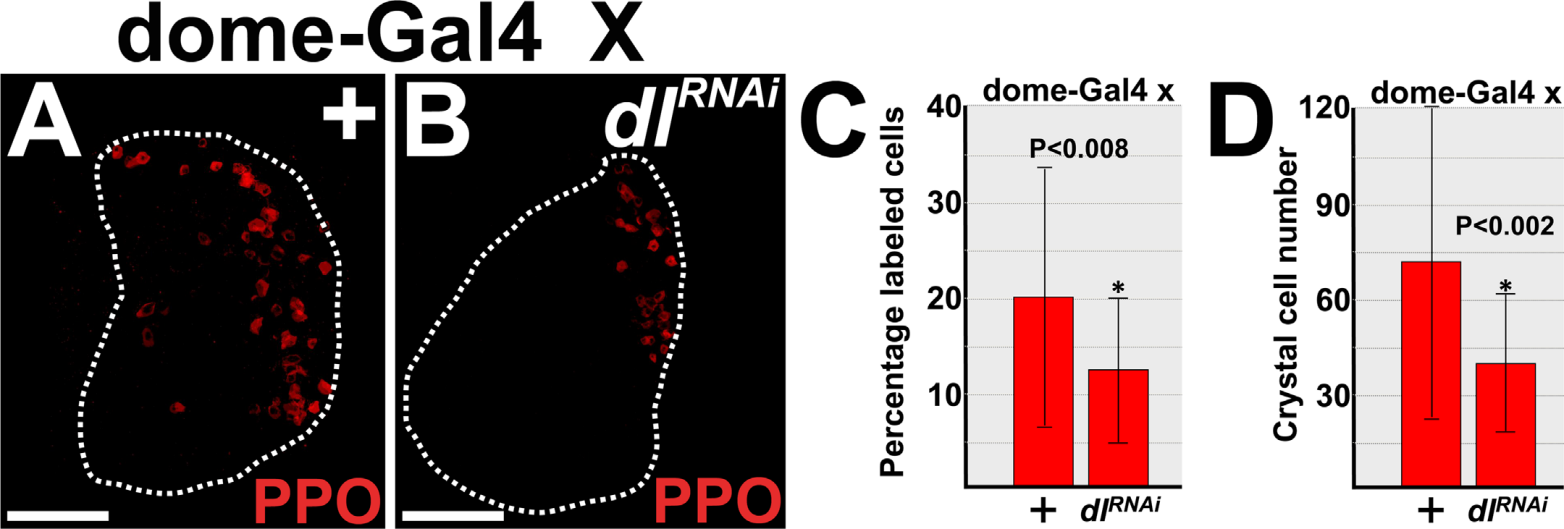
Prohemocyte-specific knockdown of Dorsal limits crystal cell differentiation. (**A,B**) Crystal cell numbers were reduced in the lymph glands of *dome-Gal4* driven *UAS-dl*^*RNAi*^ (*dl*^*RNAi*^) compared to controls (+). Lymph glands from mid-third instar larvae. White dotted lines delineate the entire lymph gland. Scale bars: 50 μm. (**C**) Histogram showing that the percentage of crystal cells decreased in *dl*^*RNAi*^ lymph glands compared to controls. (**D**) Histogram showing the number of crystal cells decreased in *dl*^*RNAi*^ lymph glands compared to controls. (**C,D**) Student’s t-test; error bars show standard deviation; control and *dl*^*RNAi*^ (n = 28); P value is as shown.

Previous studies have shown that either Dorsal or Ush can regulate crystal cell production [22,48,50,65]. Over-expression of Dorsal leads to increased lamellocyte differentiation and decreased crystal cell number [22]. This phenotype is similar to the Ush hypomorphic phenotype [50], and is consistent with our data that showed over-expression of Dorsal represses Ush protein levels. On the other hand, loss of both Dorsal and Dif function increased crystal cell number [22]. This result appeared to conflict with our data that showed Dorsal functions in prohemocytes to reduce Ush levels (Fig 2) and that Ush limits crystal cell production [48,50,65]. In an attempt to resolve this issue, we tested if knockdown of Dorsal in prohemocytes produced an increase in crystal cell number. Knockdown of Dorsal significantly reduced cell number compared to the control (Fig 5), which is in accordance with our current and previous findings. On the other hand, knockdown of Dif in prohemocytes had no effect on crystal cell number (S5 Fig). This is consistent with our data that shows knockdown of Dif had no effect on MZ size or Ush expression and supports our hypothesis that Dif does not function in prohemocytes.

**Fig 6:**
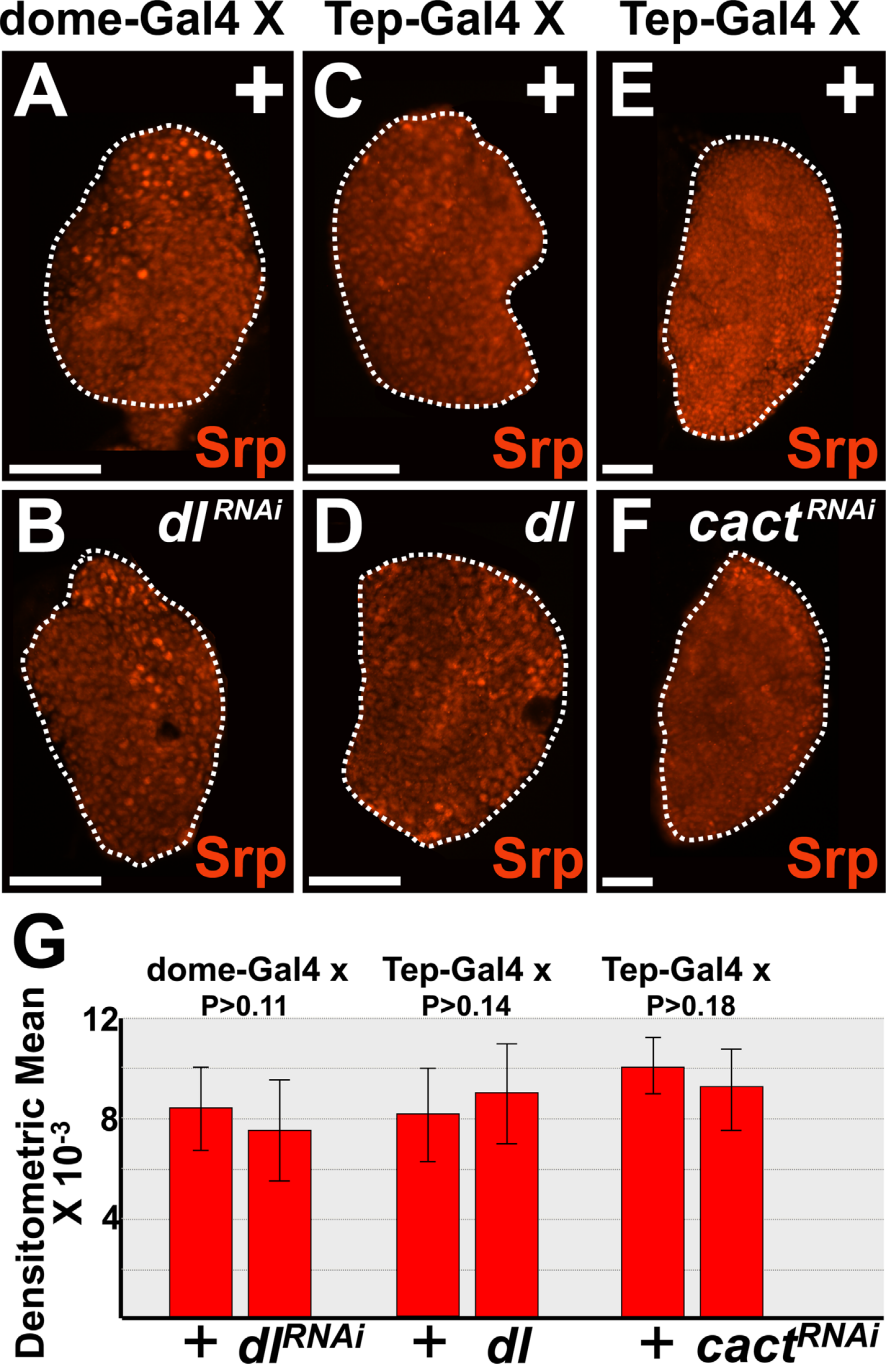
Dorsal has no effect on Srp expression. (**A-G**) Prohemocyte-specific expression of *UAS*-*dl*^*RNAi*^, *UAS*-*dl*, or *UAS-cact*^*RNAi*^ transgenes. (**A,B,G**) *dome-Gal4* or (**C-G**) *Tep-Gal4* females were crossed to control (+) males or males that carry *UAS*-*dl*^*RNAi*^, *UAS*-*dl*, or *UAS-cact*^*RNAi*^ transgenes. (**A,B**) knockdown of Dorsal, (**C,D**) over-expression of Dorsal or (**E,F**) knockdown of Cact in prohemocytes has no effect on the level of Srp expression in the lymph glands of mid-third instar larvae. White dotted lines delineate the entire lymph gland. Scale bars: 50 μm. (**G**) Histogram showing that Srp expression is not significantly different in lymph glands with altered levels of Dorsal or Cact compared to controls. Student’s t-test; for both *dome>dl*^*RNAi*^ and *Tep>cact*^*RNAi*^ and their respective controls (n = 12), control and *Tep>dl* (n = 13); error bars show standard deviation; P values are as shown.

The GATA factor, Srp, is expressed throughout the lymph gland and is required to promote prohemocyte differentiation [51,66,67]. However, Srp is also required to maintain the prohemocyte pool. In this capacity, Srp both upregulates the *ush* gene and binds to the Ush protein to form a complex that blocks prohemocyte differentiation [50,51,64]. These observations prompted us to ask if Cact and Dorsal control the level of Ush by regulating Srp expression. To test this hypothesis, we used the UAS/Gal4 system to express *UAS-dl* and *UAS-cact* transgenes in prohemocytes. Over-expression of Dorsal or knockdown of either Cact or Dorsal did not alter Srp expression levels (Fig 6), which indicates that Dorsal and Cact control Ush levels by a mechanism that does not involve regulating Srp expression.

### Toll pathway antagonist, Lwr, interacts with Ush to block differentiation and proliferation

**Fig 7:**
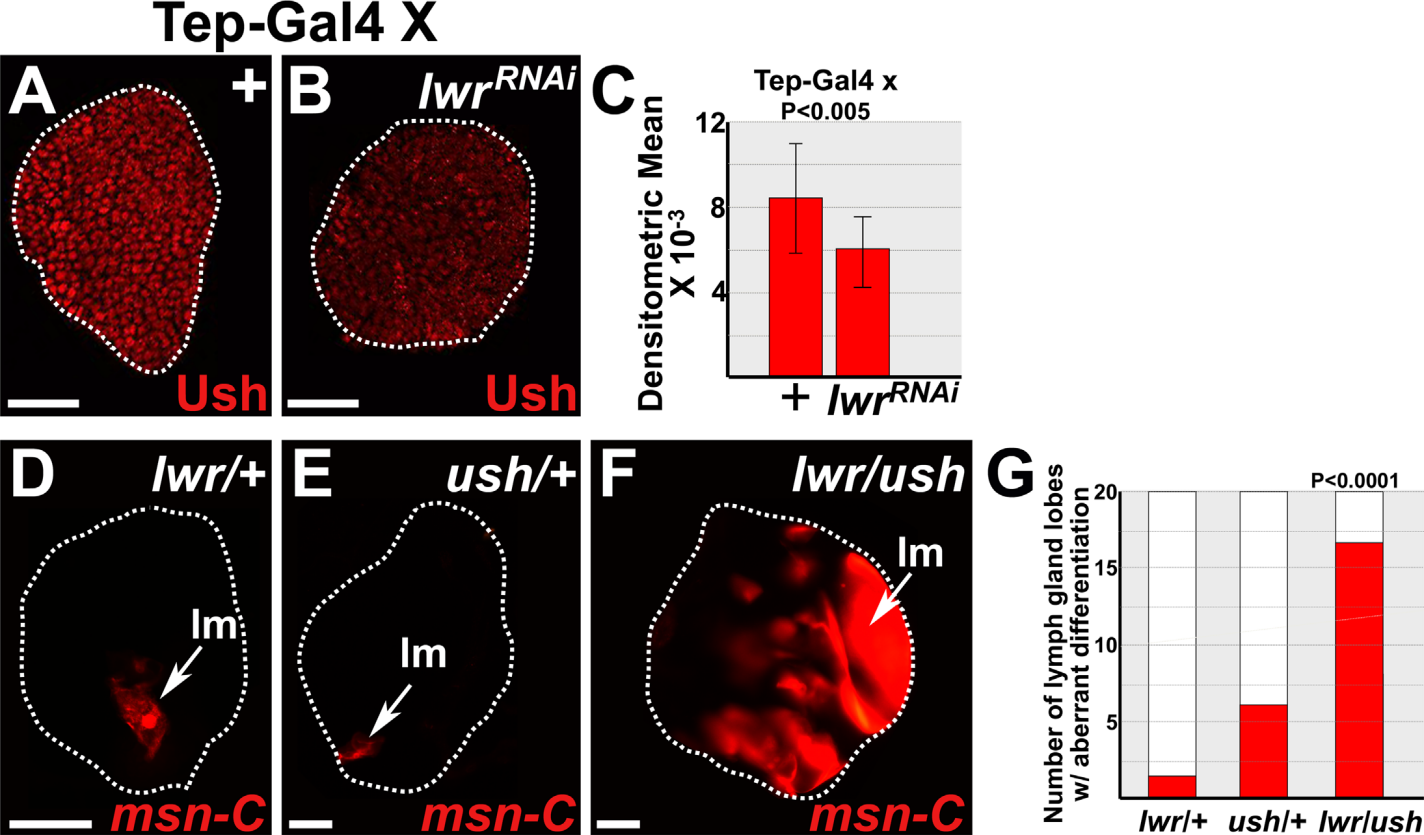
Lwr maintains Ush to block lamellocyte differentiation. (**A-C**) Lwr maintains the level of Ush expression. *Tep-Gal4* females were crossed to control (+) males or males that carry *UAS*-*lwr*^*RNAi*^ trangenes. (**B**) Knockdown of Lwr (*lwr*^*RNAi*^) in prohemocytes significantly reduced Ush levels compared to (**A**) controls (+) in the lymph glands of early-third instar larvae. (**C**) Histogram showing that the level of Ush expression is significantly reduced in lymph glands in which Lwr was knocked down compared to controls. Student’s t-test; control and *lwr*^*RNAi*^ (n = 16); error bars show standard deviation; P value is as shown. (**D-G**) Ush and Lwr act together to block lamellocyte differentiation in late-third instar larvae. (**F**) Lamellocyte differentiation in *lwr/ush* trans-heterozygous lymph glands was significantly increased compared to either (**D**) *lwr/+* or (**E**) *ush/+* lymph glands. Lamellocytes are detected using the specific marker, *MSNF9mo-DsRed* [69]. Arrows mark lamellocytes (lm). (**G**) Histogram showing that the number of primary lymph gland lobes with aberrant lamellocyte differentiation was significantly greater in *lwr/ush* transheterozygous lymph glands compared to either *lwr/+* or *ush/+* lymph glands. Chi-square test; *lwr/ush*, *lwr/+*, and *ush/+* (n = 20); P value is as shown. White dotted lines delineate the entire lymph gland. Scale bars: (**A,B**) 25 μm; (**D-F**) 50 μm.

Our studies show that Dorsal represses Ush expression levels. These findings predict that Toll antagonists, such as Lwr, should maintain Ush expression. Lwr is a small ubiquitin-like modifier (SUMO) conjugase, which functions in a variety of biological processes. Notably, Lwr restricts Toll signaling by limiting the transcriptional activity of Dorsal and maintaining the level of Cact [35,45,53]. Furthermore, like Ush, loss of Lwr function leads to loss of prohemocytes and increased lamellocyte differentiation [68]. To test if Lwr is required to maintain Ush, we used the UAS/Gal4 system coupled with RNAi technology to knockdown Lwr in prohemocytes and assessed Ush levels. Under these conditions, we observed a statistically significant decrease in Ush levels (Fig 7A–7C). Thus, Lwr is required to maintain Ush expression. We then asked if Ush and Lwr interact to control lamellocyte differentiation. To test this hypothesis, we assessed lamellocyte differentiation in *lwr/ush* trans-heterozygotes. We observed a significant increase in lamellocyte differentiation in the lymph glands of trans-heterozygotes compared to those that were singularly heterozygous for either *lwr* or *ush* (Fig 7D–7G). Thus, Lwr interacts with Ush to control lamellcyte differentiation.

**Fig 8:**
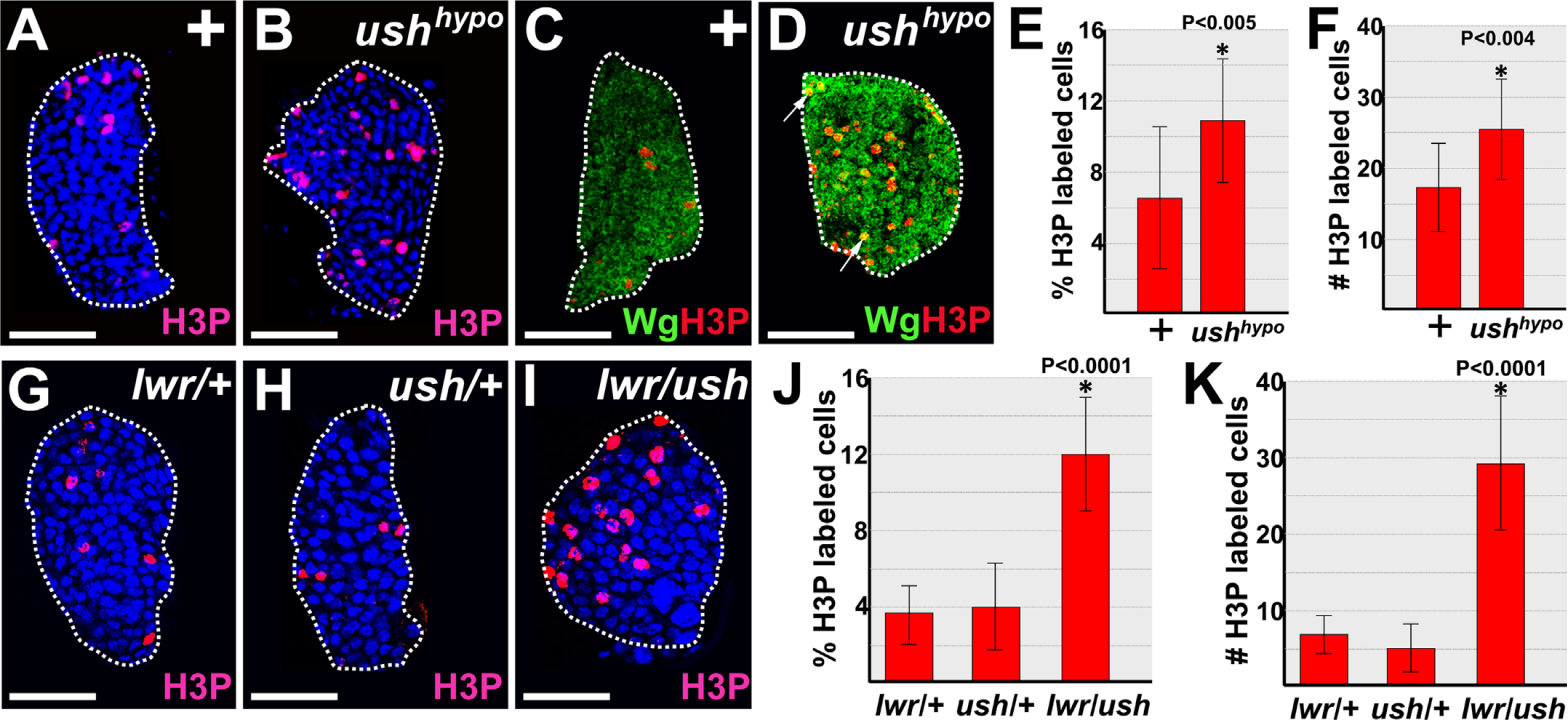
Ush and Lwr block proliferation in lymph glands from early-third instar larvae. (**A-F**) Ush limits proliferation in early-third instar lymph glands. (**A,B**) Proliferation as measured with phosphohistone H3 (H3P) antibody staining was significantly increased in (**B**) *ush*^*vx22/r24*^ hypomorphic (*ush*^*hypo*^) lymph glands compared to (**A**) controls (+). (**C)** control and **(D**) *ush*^*hypo*^ lymph glands co-stained with H3P and the prohemocyte marker, Wingless (Wg). Arrows mark cells that co-express Wg and H3P. (**E,F**) Histograms showing (**E**) the percentage of H3P labeled cells or (**F**) the number of H3P labeled cells were significantly increased in *ush*^*hypo*^ lymph glands compared to controls. Student’s t-test; error bars show standard deviation; control and *ush*^*hypo*^ (n = 14); P value is as shown. (**G-K**) Ush and Lwr act together to limit proliferation in early-third instar lymph glands. (**I**) Proliferation as measured with H3P antibody staining was significantly increased in *lwr/ush* trans-heterozygous lymph glands compared to either (**G**) *lwr/+* or (**H**) *ush/+* lymph glands. (**J,K**) Histograms showing (**J**) the percentage of H3P labeled cells or (**K**) the number of H3P labeled cells were significantly increased in *lwr/ush* trans-heterozygous lymph glands compared to either *lwr/+* or *ush/+* lymph glands. One way Anova; error bars show standard deviation; *lwr/ush* trans-heterozygous lymph glands compared to either *lwr/+*, *ush/+*and *lwr/ush* (n = 17); P value is as shown. White dotted lines delineate the entire lymph gland. Scale bars: 25 μm.

Lwr maintains prohemocytes in a state of proliferative quiescence during the third larval instar [68]. Given that Lwr is required for Ush expression, we tested if loss of Ush function increased proliferation by assessing phosphohistone H3 (H3P) expression in the lymph glands of *ush* hypomorphs (*ush*^*vx22*^*/ush*^*r24*^) and wild-type controls. During the early-third larval instar, H3P-positive cells increased significantly in *ush* hypomorphs compared to controls (Fig 8A–8F). Early-third larval instar lymph glands contain primarily prohemocytes and some of the H3P-positive cells also expressed the prohemocyte marker, Wingless (Wg) [18] (Fig 8C and 8D). This indicated that these proliferating cells were prohemocytes. We then determined if Ush interacts with Lwr to promote proliferative quiescence in early-third instar larvae. H3P-positive cells increased significantly in the lymph glands of *lwr/ush* trans-heterozygotes compared to those that were singularly heterozygous for either *ush* or *lwr* (Fig 8G–8K). Thus, Ush interacts with Lwr to promote proliferative quiescence during the early-third larval instar.

Lwr is required to maintain proliferative quiescence until at least the mid-third larval instar [22]. In contrast, there was no significant increase in the number of H3P-positive cells in lymph glands from either *ush* hypomorphs [50,52] or larvae in which Ush was knocked down in the MZ (S6 Fig) during this larval stage. Likewise, there was no change in the number of H3P-positive cells in lymph glands from larvae in which either Cact or Dorsal expression was altered in the MZ (S6 Fig). Prohemocytes have been shown to transition from rapid proliferation to quiescence during the interval between the late-second to early-third larval instar [6]. Thus, Ush may interact with Lwr to establish proliferative quiescence during the early-third instar. Once quiescence is established, Lwr most likely acts in the absence of Ush to maintain prohemocyte quiescence.

## Discussion

In this report we describe novel functions for Dorsal and Cact as intrinsic regulators of lymph gland MZ prohemocytes. We showed that knockdown of Dorsal increased the size of the MZ and that knockdown Cact, reduced the size of the MZ. The change in MZ size may have resulted from changes in cell size rather than cell number. However, this seems unlikely given that lymph glands with increased MZ size also had increased numbers of prohemocytes and those with decreased MZ size also had decreased numbers of prohemocytes. Thus, the observed change in MZ size most likely results from changes in the number of prohemocytes. Overall, our findings indicate that antagonism between Dorsal and Cact regulates prohemocyte number during steady state conditions.

We also showed that upregulation of Dorsal in prohemocytes promotes lamellocyte differentiation, whereas loss of Cact increased lamellocyte differentiation. This suggests that Dorsal and Cact regulate prohemocyte population size by controlling the choice between multipotency and differentiation. Furthermore, our studies indicate that this involves regulating the level of Ush. Importantly, we showed that Ush is a downstream target of Dorsal and antagonism between Ush and Dorsal regulates prohemocyte differentiation during steady state conditions. Thus, Dorsal repression of Ush may prime prohemocytes for rapid entrance into the differentiation pathway. Conversely, Ush antagonizes Dorsal activity and this prevents excessive prohemocyte loss. This proposed model describes how antagonism between an inflammation signaling pathway and a key master regulator can control prohemocyte differentiation during steady state conditions.

**Fig 9:**
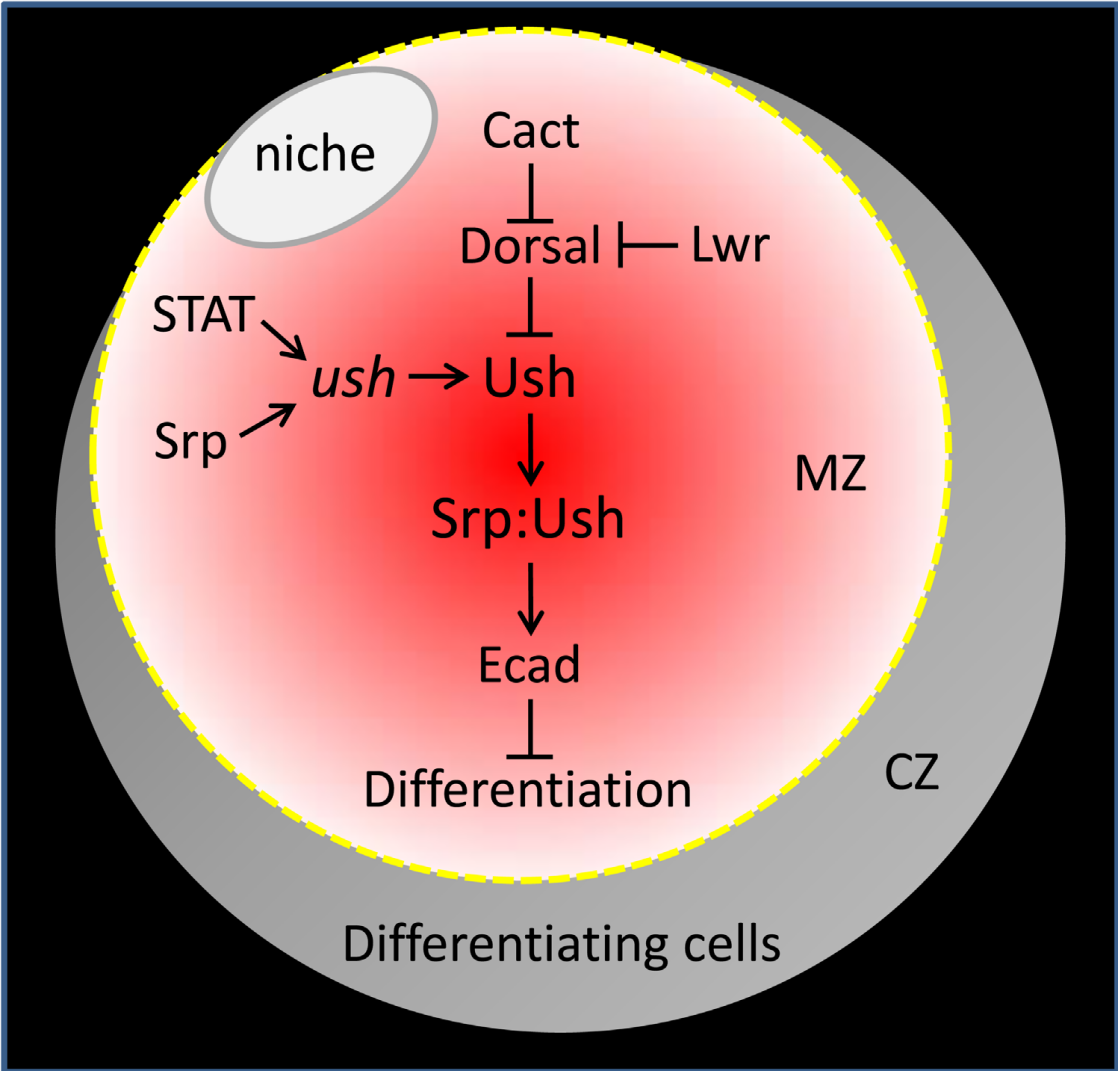
Model of prohemocyte regulation during steady state hematopoiesis. Cact and Lwr antagonize Dorsal activity [33–35,45,53], whereas Dorsal represses Ush protein expression. STAT and Srp bind to the *ush CRM* to activate *ush* gene expression and thereby maintain Ush protein levels [50,64]. Ush binds to Srp to form a complex that maintains E-cadherin (Ecad) expression. Ecad is required to maintain prohemocyte multipotency and block differentiation [51]. Niche (PSC) in white, MZ prohemocytes are in shades of red, differentiating CZ cells are in shades of grey. The model is a simplification of the total genetic complexity that regulates the prohemocyte population during steady state conditions and does not list all components that control the choice between multipotency and differentiation.

The molecular mechanism by which Dorsal downregulates Ush in the lymph gland is not known. Gene expression profiling of early stage embryos show that the level of *ush* transcript increased at least 6-fold in animals harboring Toll signaling mutations [70]. This observation, coupled with our identification of consensus Dorsal binding sites within the *ush CRM*, suggested that Dorsal downregulates Ush by repressing gene expression. However, we observed that *ush CRM-reporter lacZ* activity was not repressed by Dorsal. It is possible that the *CRM-reporter lacZ* transgenes lack elements that would make them responsive to repression by Dorsal. However, this seems unlikely given that none of the five *CRM-reporter lacZ* transgenes were repressed by Dorsal, including the 7.5 kb CRM that recapitulates *ush* expression in most embryonic tissues and in the lymph gland [64]. Alternatively, Dorsal may not repress Ush protein levels by downregulating gene expression, either directly by binding to the consensus sites within the *ush* CRM or indirectly by regulating other factors that bind the CRM. Two factors, Srp and STAT, have been shown to activate the 150 bp minimal *ush CRM-reporter lacZ*. Given that CRM activity was not repressed by Dorsal, it is unlikely that Dorsal represses Ush expression by inhibiting the function of either of these factors. Furthermore, Srp levels are not affected by altering the expression of either Dorsal or Cact in the MZ, which is consistent with this hypothesis. Although the precise mechanism by which Dorsal represses Ush is unknown, it is clear that all three factors (Dorsal, STAT and Srp) provide molecular inputs that ultimately determine Ush protein levels during steady state conditions. This regulates GATA:FOG complex formation, which maintains E-cadherin in prohemocytes in order to promote multipotency and limit differentiation [51]. Thus, Ush appears to be a key node that integrates the input from various signaling pathways to control prohemocyte differentiation (Fig 9).

Dorsal and Dif have overlapping functions during steady state hematopoiesis and the immune response. However, our studies indicate that unlike Dorsal, Dif does not function in prohemocytes. We tested three different biological endpoints, including MZ size, crystal cell differentiation and Ush expression and showed that knockdown of Dif in prohemocytes did not affect any of these parameters. These observations indicate that Dorsal acts independently of Dif to downregulate Ush and limit the size of the prohemocyte pool. Previous studies have shown that Dorsal functions during embryogenesis to regulate dorsal/ventral patterning [30]. However, there is no evidence that Dif functions in these cells [71]. Likewise, our studies show that Dorsal functions in undifferentiated prohemocytes, whereas Dif does not appear to function in these cells. Thus, Dorsal is an important regulator of both undifferentiated and differentiated blood cells, whereas Dif may primarily regulate blood cells that are in the later stages of development.

We showed that knockdown of Dorsal in prohemocytes reduced the number of crystal cells. This observation supports our hypothesis that Dorsal promotes prohemocyte differentiation. Furthermore, this finding is consistent with our previous studies that showed Ush limits crystal cell differentiation and our new findings that showed Dorsal downregulates Ush. On the other hand, a recent study showed that systemic loss of both Dorsal and Dif increased the number of crystal cells. One possible explanation for these seemingly contradictory results is that Dorsal might act with Dif later in hematopoiesis to limit the number of committed crystal cell precursors. In these more advanced cells, Dif may convert Dorsal from a prohemocyte differentiation activator to a crystal cell inhibitor. Interactions between transcriptional regulators that promote changes in function have been observed in other gene regulatory contexts. For example, Srp interacts with Lozenge to promote crystal cell differentiation. In contrast, when Srp is bound to Ush, it inhibits crystal cell differentiation [65]. It should be noted that neither over-expression of Ush nor knockdown of Dorsal completely blocks prohemocyte differentiation or crystal cell production ([48,72] and this study). Thus, while loss of Dorsal function may limit differentiation of the more primitive prohemocyte population, loss of both Dorsal and Dif in more advanced crystal cell precursors may enlarge the size of this remnant population leading to a net increase in the number of crystal cells.

Lwr limits prohemocyte proliferation during the mid- to late- third larval instar and this has been linked to its ability to block lamellocyte differentiation [22]. In contrast, we showed that Ush, Dorsal and Cact do not appear to regulate prohemocyte proliferation during this developmental period despite their capacity to control lamellocyte differentiation. Notably, the cell cycle inhibitor, Decapo, is downregulated in *lwr* mutants [22]. However, Decapo is not downregulated in mid- to late-third instar *ush* mutants (Gao and Fossett, unpublished), providing further support for the notion that Ush does not function with Lwr to control the cell cycle during this developmental stage. Our work shows Lwr maintains Ush and previous studies show Lwr blocks Dorsal activity by maintaining Cact protein levels [35]. Furthermore, we showed that Lwr interacts with Ush to block lamellocyte differentiation. Collectively, these observations suggest that Lwr blocks lamellocyte differentiation by at least two different mechanisms. In the first case, Lwr upregulate**s** Decapo, which limits proliferation. This limits lamellocyte differentiation and microtumor formation [22]. In the second case, Lwr inhibits Dorsal transcriptional activity [35,45,53], which maintains Ush expression to block lamellocyte differentiation. It is not surprising that Lwr controls lamellocyte differentiation through at least two different mechanisms. Lwr is the *Drosophila* homolog of Ubc9, a small ubiquitin-like modifier (SUMO) conjugase [73]. This enzyme plays an essential role in protein post-translational sumolylation, which regulates a number of process that likely control prohemocyte differentiation including chromatin organization, cell cycle regulation and signal transduction [73].

Srp binds Ush and forms a GATA:FOG complex that blocks lamellocyte differentiation to maintain the prohemocyte pool. Conversely, increasing the level of unbound Srp in prohemocytes drives lamellocyte differentiation [51]. Thus, the ratio of bound to unbound Srp determines the choice between multipotency and differentiation. However, unbound Srp upregulates *ush* expression and increases GATA:FOG complex formation, which limits prohemocyte differentiation. Indeed, our previous work showed that over-expressing wild-type Srp produced lamellocyte differentiation; however, differentiation was less than that produced by over-expressing a mutant version of Srp (Srp^V421G^) that cannot bind Ush [51]. Alternatively, decreasing the level of Ush both limits GATA:FOG complex formation and increases the level of unbound Srp. Reactive oxygen species (ROS) upregulate Srp expression [74], whereas Dorsal had no effect on Srp but did downregulate Ush. Thus, Dorsal should produce a more pronounced prohemocyte inflammatory response than increased ROS. In support of this hypothesis, over-expression of Dorsal produced robust lamellocyte differentiation that was comparable to over-expression of the Srp^V421G^ mutant. During stress-response, multiple signaling pathways are activated, which may act in combination to control GATA:FOG complex formation as a means to optimize the level of hematopoietic progenitor differentiation.

Previous work using mammalian systems have shown that activation of NFκB in murine HSPCs drives differentiation. Furthermore, a growing body of evidence suggests that inflammatory signaling pathways are important for proper maintenance of hematopoietic progenitors during steady-state conditions [1]. For example, murine hematopoietic stem cells (HSCs) that lack the TNF receptor p55 (TNFR1) have impaired self-renewal and reduced engraftment capacity with age [75,76]. Moreover, loss of TLR4 produces increased numbers of quiescent HSCs [77]. *Drosophila* prohemocytes share key characteristics with mammalian HPSCs, including quiescence, multipotency and niche-responsiveness. Thus, these cells can serve as a model to identify key HSPC regulators. In this regard, our work showed that direct activation of hematopoietic progenitors by NF-κB is an evolutionarily conserved mechanism. Moreover, our findings extend the knowledge of NF-κB-directed progenitor differentiation by showing that the FOG homolog, Ush, antagonizes Dorsal-driven prohemocyte loss. This may be an important regulatory mechanism given that FOG-1 and NF-κB are expressed in mammalian HSPCs [5,54]. Thus, this prohemocyte regulatory node provides an opportunity to use *Drosophila* to investigate how inflammatory signaling pathways regulate HSPCs during steady-state hematopoiesis.

## Supporting Information

S1 Fig Schematic of the third larval instar lymph gland. Primary (1^0^), secondary (2^0^) lobes and the relative positions of the three domains within the 1^0^ lobe are shown. The cortical zone (CZ) is depicted in shades of grey, the medullary zone (MZ) is depicted in shades of red, and the stem cell niche (PSC; Posterior Signaling Center) is depicted in white. Prohemocytes reside in the MZ. Differentiating cells reside in the CZ.
(TIF)

S2 Fig Prohemocyte function of Toll pathway members Dorsal, Cact and Dif. (**A-C**) Knockdown of Dorsal (*dl*^*RNAi*^) significantly reduced Dorsal protein levels compared to controls (+). *dome-Gal4* females were crossed to control (+) males or males that carry *UAS*-*dl*^*RNAi*^ transgene. (**C**) Histogram showing that Dorsal expression was significantly reduced in *dome-Gal4* driven *UAS-dl*^*RNAi*^ lymph glands compared to controls. Student’s t-test; error bars show standard deviation; P value is as shown; control and *dl*^*RNAi*^ (n = 17). (**D,E**) Loss of Dif function has no effect on the size of the prohemocyte pool. (**D**) Histogram showing that the size of the MZ did not change in lymph glands in which Dif was knocked down in prohemocytes. Student’s t-test; error bars show standard deviation; P values are as shown; control and *Dif* ^*RNAi*^ (n = 15). (**E**) Histogram showing the size of the MZ did not change in *Dif* heterozygous lymph glands compared to controls. Student’s t-test; error bars show standard deviation; P values are as shown control and *Dif/+* (n = 17). (**F-H**) Knockdown of Cact (*cact*^*RNAi*^) significantly reduced Cact protein levels compared to controls (+). *Tep-Gal4* females were crossed to control (+) males or males that carry *UAS*-*cact*^*RNAi*^ transgene. (**H**) Histogram showing that Cact expression was significantly reduced in *Tep-Gal4* driven *UAS-cact*^*RNAi*^ lymph glands compared to controls. Student’s t-test; error bars show standard deviation; P value is as shown; *cact*^*RNAi*^ and controls (n = 15). (**I**) Histogram showing that the number of primary lymph gland lobes with aberrant lamellocyte differentiation was significantly greater in *dome-Gal4* driven *UAS-cact*^*RNAi*^ lymph glands compared to controls. Fisher’s Exact test; P value is as shown; controls, n = 18; *cact*^*RNAi*^, n = 16. (**J-M**) Cell counts in lymph glands with altered expression of Dorsal, Dif or Cact. (**J**) Histogram showing the number of Odd-expressing cells increased in lymph glands in which Dorsal was knocked down (*dl*^*RNAi*^) in the MZ. Student’s t-test; error bars show standard deviation; P values are as shown; control and *dl*^*RNAi*^ (n = 8). (**K**) Histogram showing the number of Odd-expressing cells decreased in lymph glands in which Dorsal was over-expressed. Student’s t-test; error bars show standard deviation; P values are as shown; control and *dl* (n = 6). (**L**) Histogram showing knock down of Dif had no effect on the number of Odd-expressing cells. Student’s t-test; error bars show standard deviation; control and *Dif*^*RNAi*^ (n = 6); P values are as shown. (**M**) Histogram showing the number of Odd-expressing cells decreased in lymph glands in which Cact was knocked down (*cact*^*RNAi*^). Student’s t-test; error bars show standard deviation; P values are as shown; control and *cact*^*RNAi*^ (n = 6). White dotted lines delineate the entire lymph gland. Scale bars: 50 μm. (**A-H, J-M**) lymph glands from mid-third instar larvae; (**I)** lymph glands from late-third instar larvae. (**N**) Model depicting Cact and Dorsal regulation of prohemocyte choice between multipotency and differentiation. MZ prohemocytes are depicted in red, CZ differentiating cells in grey and the PSC (niche) in white. Cact antagonizes Dorsal function to maintain prohemocyte multipotency and block differentiation. Loss of Cact leads to increased Dorsal activation and prohemocyte differentiation.
(TIF)

S3 Fig Activity of the 7.5 kb *ush CRM- lacZ* transgene does not increase in *dl* heterozygotes. (**A**) Control larvae with one copy of the 7.5 kb *ush CRM- lacZ* transgene. (**B**) *dl* heterozygous larvae with one copy of the 7.5 kb *ush CRM- lacZ* transgene. Scale bars: 50 μm. White dotted lines delineate entire lymph gland. (**C**) Histogram showing the level of β-galactosidase expression is not significantly different between *dl/+* and controls. Student’s t-test; error bars show standard deviation; P value is as shown; control and *dl*/+ (n = 12).
(TIF)

S4 Fig Ush antagonizes Dorsal-driven prohemocyte loss. **(A)** Histogram showing the number of Odd-expressing cells increased in lymph glands in which Ush was over-expressed (*ush*^*GOF*^) in the MZ. Student’s t-test; error bars show standard deviation; P values are as shown; control and *ush*^*GOF*^ (n = 6). (**B**) Histogram showing the number of Odd-expressing cells increased in lymph glands with *Tep-Gal4* driven co-expression of *UAS*-*ush* and *UAS-cact*^*RNAi*^ compared to *Tep-Gal4* driven expression of *UAS*-*cact*^*RNAi*^ alone. Student’s t-test; error bars show standard deviation; P values are as shown; *ush* with *cact*^*RNAi*^ and *cact*^*RNAi*^ alone (n = 8). (**C**) Histogram showing the number of Odd-expressing cells increased in *dl/ush* lymph glands compared to *ush/+* lymph glands. Student’s t-test; error bars show standard deviation; P values are as shown; *ush/+* and *dl/ush* (n = 6). (**D**) Model illustrating Cact and Dorsal regulation of Ush controls prohemocyte choice between multipotency and differentiation. MZ prohemocytes are depicted in red, CZ differentiating cells in grey and the PSC in white. Cact antagonizes Dorsal function to maintain the level of Ush, which maintains prohemocyte multipotency and blocks differentiation. Loss of Cact leads to increased Dorsal activation, which reduces the level of Ush and promotes prohemocyte differentiation.
(TIF)

S5 Fig Prohemocyte-specific knockdown of Dif does not affect crystal cell differentiation. (**A,B**) Crystal cell numbers were unchanged in the lymph glands from *dome-Gal4* driven *UAS-Ddl*^*RNAi*^ (*Dif*^*RNAi*^) larvae compared to controls. Lymph glands from mid-third instar larvae. White dotted lines delineate the entire lymph gland. Scale bars: 50 μm. (**C**) Histogram showing the percentage of crystal cells in controls and *Dif*^*RNAi*^ lymph glands. (**D**) Histogram showing the number of crystal cells in controls and *Dif*^*RNAi*^ lymph glands. (**C,D**) Student’s t-test; error bars show standard deviation; control and *Dif*^*RNAi*^ (n = 17); P value is as shown.
(TIF)

S6 Fig Altering the expression of *ush*, *cact* or *dl* in prohemocytes had no effect on proliferation in mid-third instar larvae. *dome-Gal4* females were crossed to control (+) males or males that carry *UAS-ush*^*RNAi*^, *UAS-cact*^*RNAi*^, *UAS*-*dl* or *UAS*-*dl*^*RNAi*^ transgenes. (**A-C**) Knockdown of Ush (*ush*^*RNAi*^), (**D-F**) knockdown of Cact (*cact*^*RNAi*^), (**G-I**) over-expression of Dorsal (*dl*^*GOF*^) or (**J-L**) knockdown of Dorsal (*dl*^*RNAi*^) had no effect on proliferation as measured with phosphohistone H3 (H3P) antibody staining. Histograms showing (**C,F,I,L**) the number of H3P labeled cells was not significantly different in lymph glands in which the expression of *ush*, *cact* or *dl* was altered in prohemocytes compared to their respective controls. Student’s t-test; error bars show standard deviation; P value is as shown; control and *ush*^*RNAi*^ (n = 24); control and *cact*^*RNAi*^ (n = 21); control and *dl*^*GOF*^ (n = 19); control and *dl*^*RNAi*^ (n = 15). Lymph glands from mid-third instar larvae. White dotted lines delineate the entire lymph gland. Scale bars: 50 μm.
(TIF)

S1 File Measurements and statistical analyses for histograms presented in figures and supporting figures. (XLSX)
