# Supplementary figures and images for: The Friend of GATA Transcriptional Co-Regulator, U-Shaped, Is a Downstream Antagonist of Dorsal-Driven Prohemocyte Differentiation in Drosophila
Source: PLoS One. 2016 May 10;11(5):e0155372. doi: 10.1371/journal.pone.0155372 (PMC4862636; doi:10.1371/journal.pone.0155372)

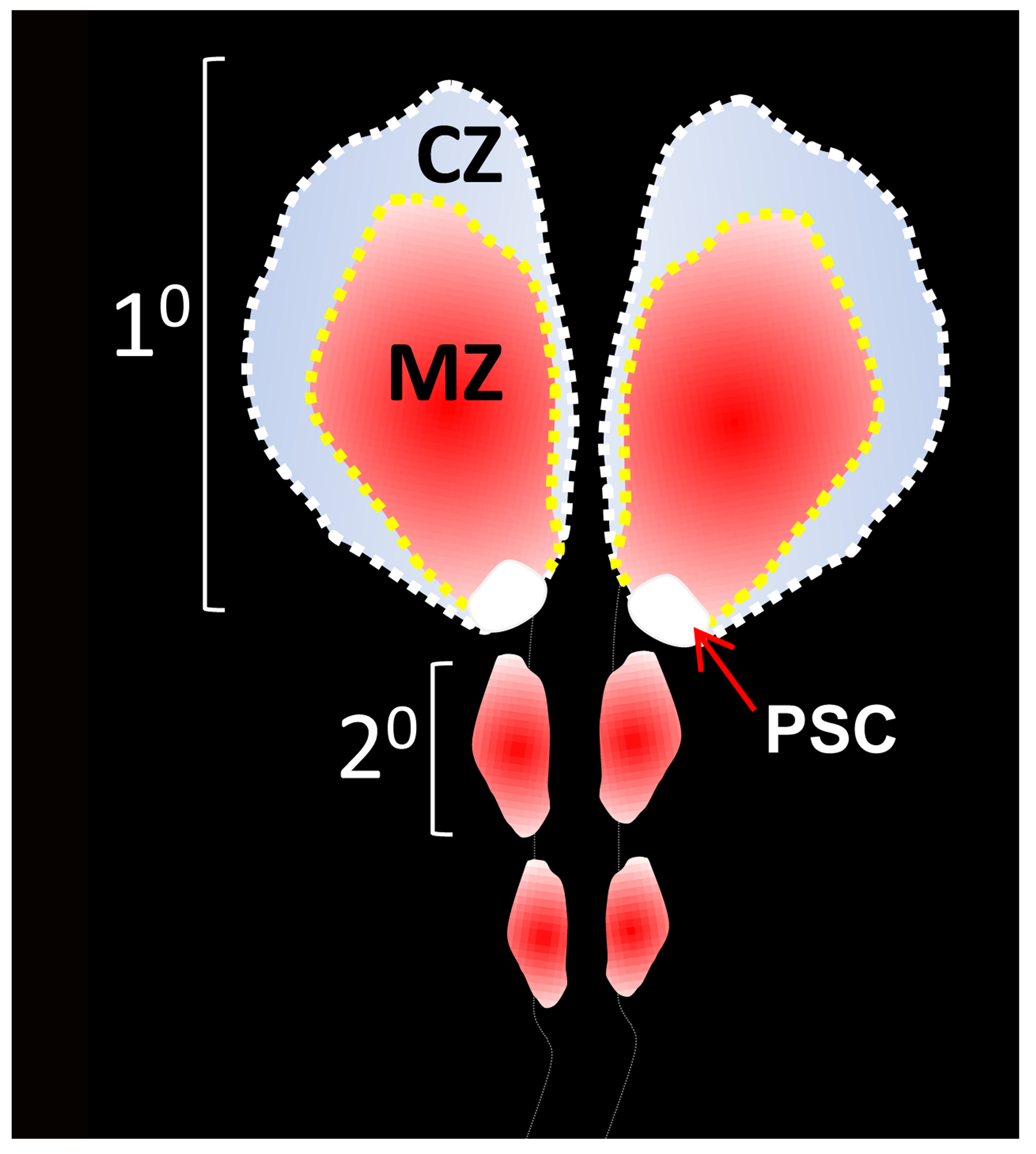

Supplement: S1 Fig — Primary (10), secondary (20) lobes and the relative positions of the three domains within the 10 lobe are shown. The cortical zone (CZ) is depicted in shades of grey, the medullary zone (MZ) is depicted in shades of red, and the stem cell niche (PSC; Posterior Signaling Center) is depicted in white. Prohemocytes reside in the MZ. Differentiating cells reside in the CZ. (TIF) [file pone.0155372.s001.tif]

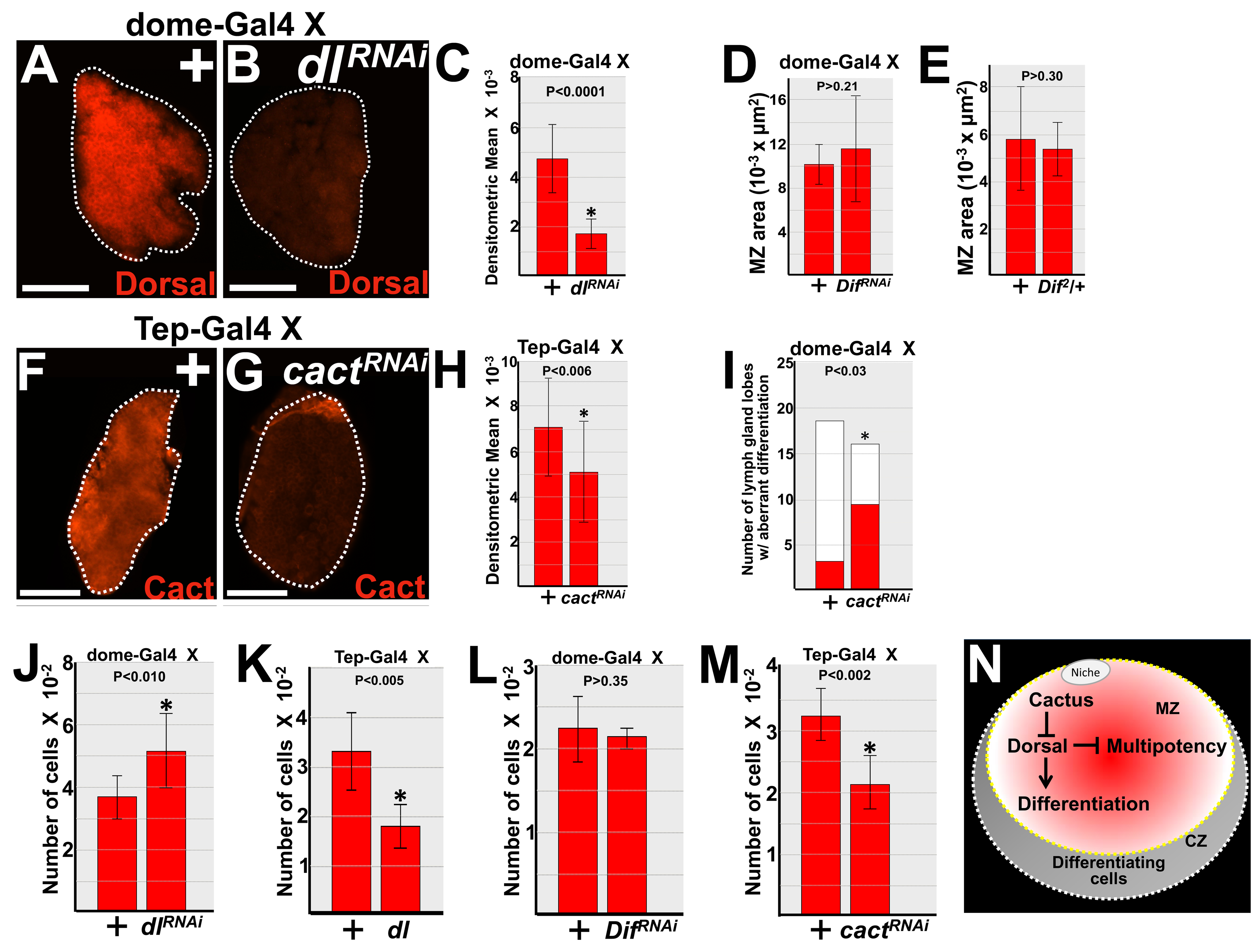

Supplement: S2 Fig — (A-C) Knockdown of Dorsal (dlRNAi) significantly reduced Dorsal protein levels compared to controls (+). dome-Gal4 females were crossed to control (+) males or males that carry UAS-dlRNAi transgene. (C) Histogram showing that Dorsal expression was significantly reduced in dome-Gal4 driven UAS-dlRNAi lymph glands compared to controls. Student’s t-test; error bars show standard deviation; P value is as shown; control and dlRNAi (n = 17). (D,E) Loss of Dif function has no effect on the size of the prohemocyte pool. (D) Histogram showing that the size of the MZ did not change in lymph glands in which Dif was knocked down in prohemocytes. Student’s t-test; error bars show standard deviation; P values are as shown; control and Dif RNAi (n = 15). (E) Histogram showing the size of the MZ did not change in Dif heterozygous lymph glands compared to controls. Student’s t-test; error bars show standard deviation; P values are as shown control and Dif/+ (n = 17). (F-H) Knockdown of Cact (cactRNAi) significantly reduced Cact protein levels compared to controls (+). Tep-Gal4 females were crossed to control (+) males or males that carry UAS-cactRNAi transgene. (H) Histogram showing that Cact expression was significantly reduced in Tep-Gal4 driven UAS-cactRNAi lymph glands compared to controls. Student’s t-test; error bars show standard deviation; P value is as shown; cactRNAi and controls (n = 15). (I) Histogram showing that the number of primary lymph gland lobes with aberrant lamellocyte differentiation was significantly greater in dome-Gal4 driven UAS-cactRNAi lymph glands compared to controls. Fisher’s Exact test; P value is as shown; controls, n = 18; cactRNAi, n = 16. (J-M) Cell counts in lymph glands with altered expression of Dorsal, Dif or Cact. (J) Histogram showing the number of Odd-expressing cells increased in lymph glands in which Dorsal was knocked down (dlRNAi) in the MZ. Student’s t-test; error bars show standard deviation; P values are as shown; control and dlRNAi [file pone.0155372.s002.tif]

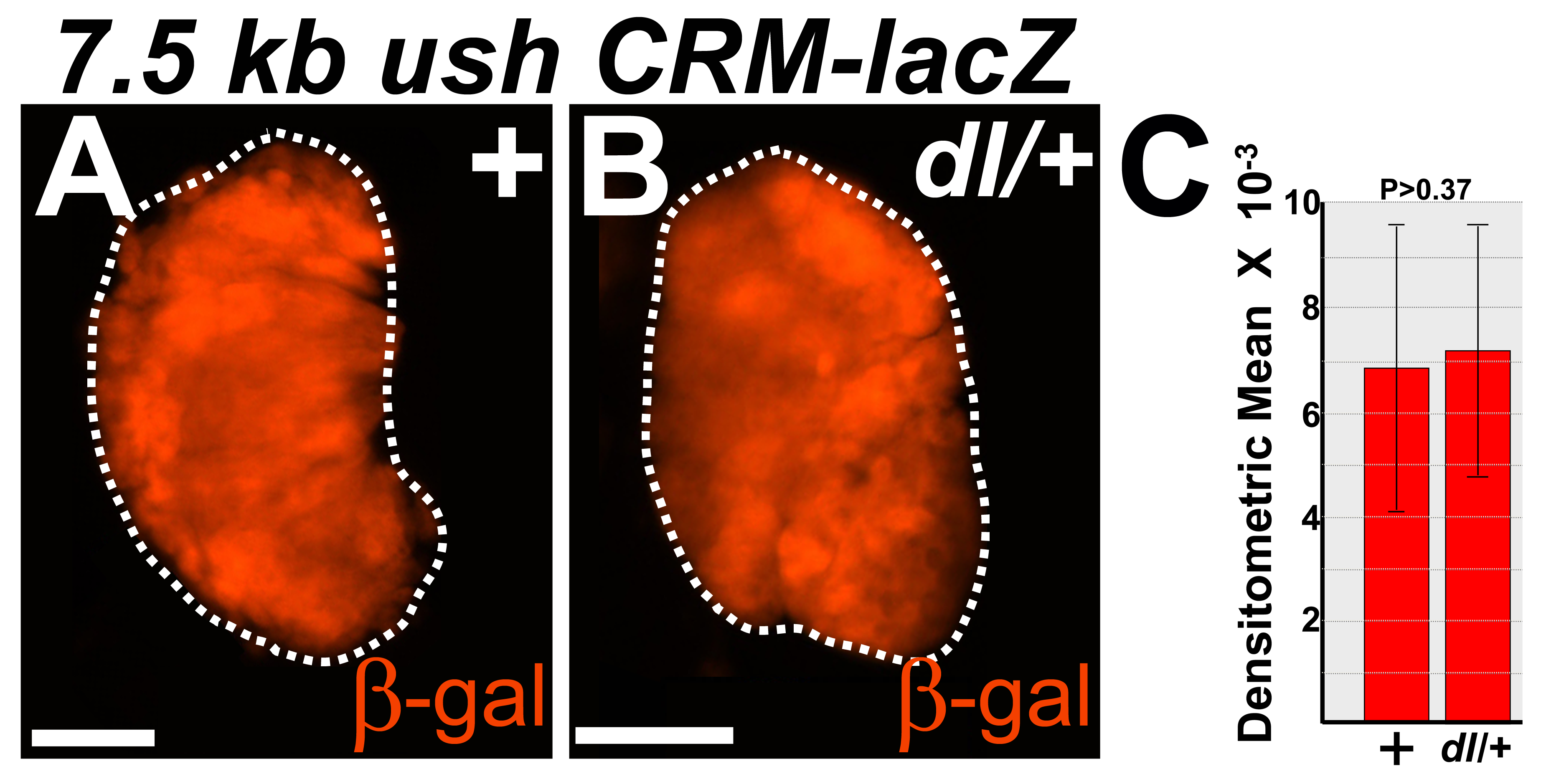

Supplement: S3 Fig — (A) Control larvae with one copy of the 7.5 kb ush CRM- lacZ transgene. (B) dl heterozygous larvae with one copy of the 7.5 kb ush CRM- lacZ transgene. Scale bars: 50 μm. White dotted lines delineate entire lymph gland. (C) Histogram showing the level of β-galactosidase expression is not significantly different between dl/+ and controls. Student’s t-test; error bars show standard deviation; P value is as shown; control and dl/+ (n = 12). (TIF) [file pone.0155372.s003.tif]

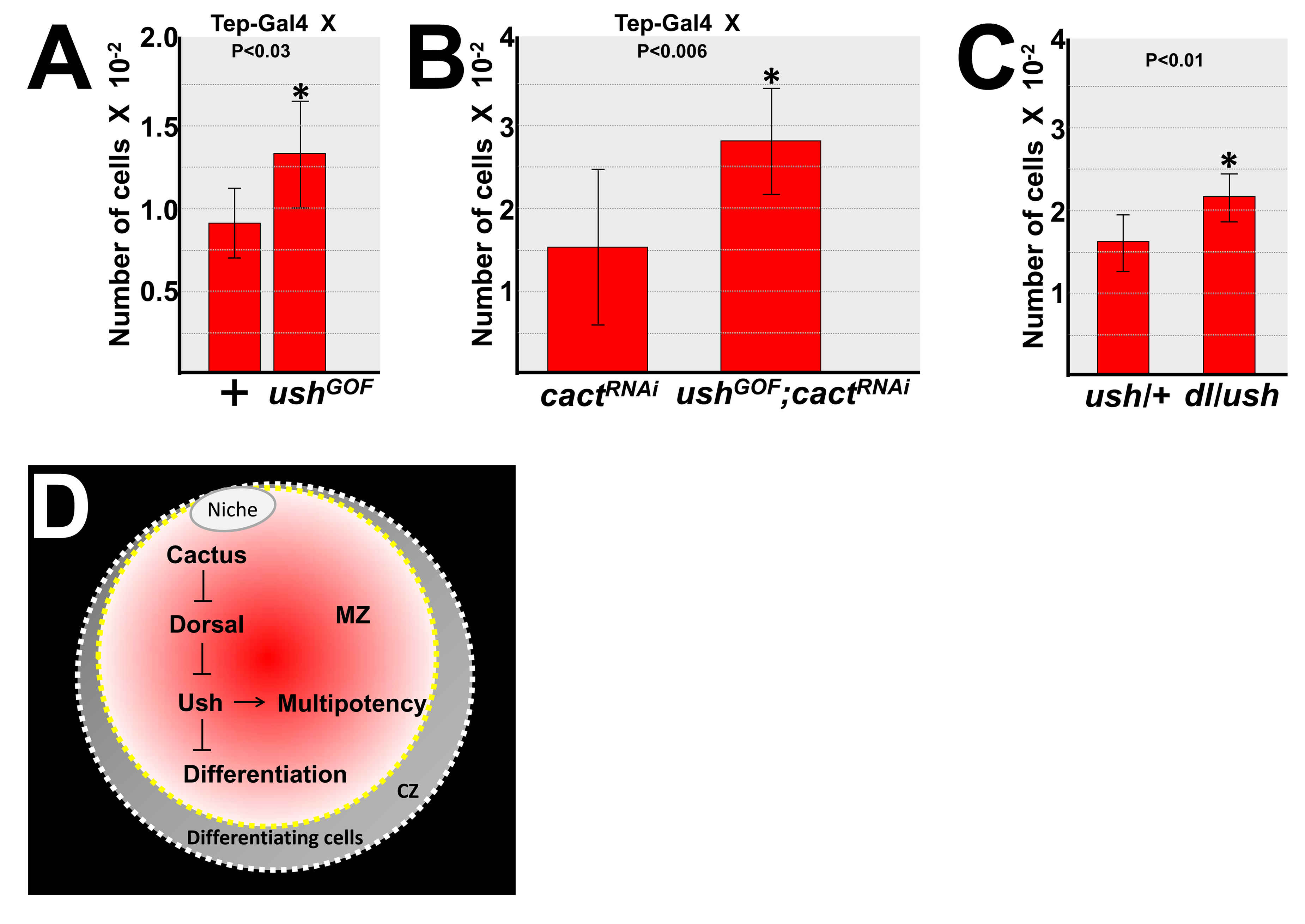

Supplement: S4 Fig — (A) Histogram showing the number of Odd-expressing cells increased in lymph glands in which Ush was over-expressed (ushGOF) in the MZ. Student’s t-test; error bars show standard deviation; P values are as shown; control and ushGOF (n = 6). (B) Histogram showing the number of Odd-expressing cells increased in lymph glands with Tep-Gal4 driven co-expression of UAS-ush and UAS-cactRNAi compared to Tep-Gal4 driven expression of UAS-cactRNAi alone. Student’s t-test; error bars show standard deviation; P values are as shown; ush with cactRNAi and cactRNAi alone (n = 8). (C) Histogram showing the number of Odd-expressing cells increased in dl/ush lymph glands compared to ush/+ lymph glands. Student’s t-test; error bars show standard deviation; P values are as shown; ush/+ and dl/ush (n = 6). (D) Model illustrating Cact and Dorsal regulation of Ush controls prohemocyte choice between multipotency and differentiation. MZ prohemocytes are depicted in red, CZ differentiating cells in grey and the PSC in white. Cact antagonizes Dorsal function to maintain the level of Ush, which maintains prohemocyte multipotency and blocks differentiation. Loss of Cact leads to increased Dorsal activation, which reduces the level of Ush and promotes prohemocyte differentiation. (TIF) [file pone.0155372.s004.tif]

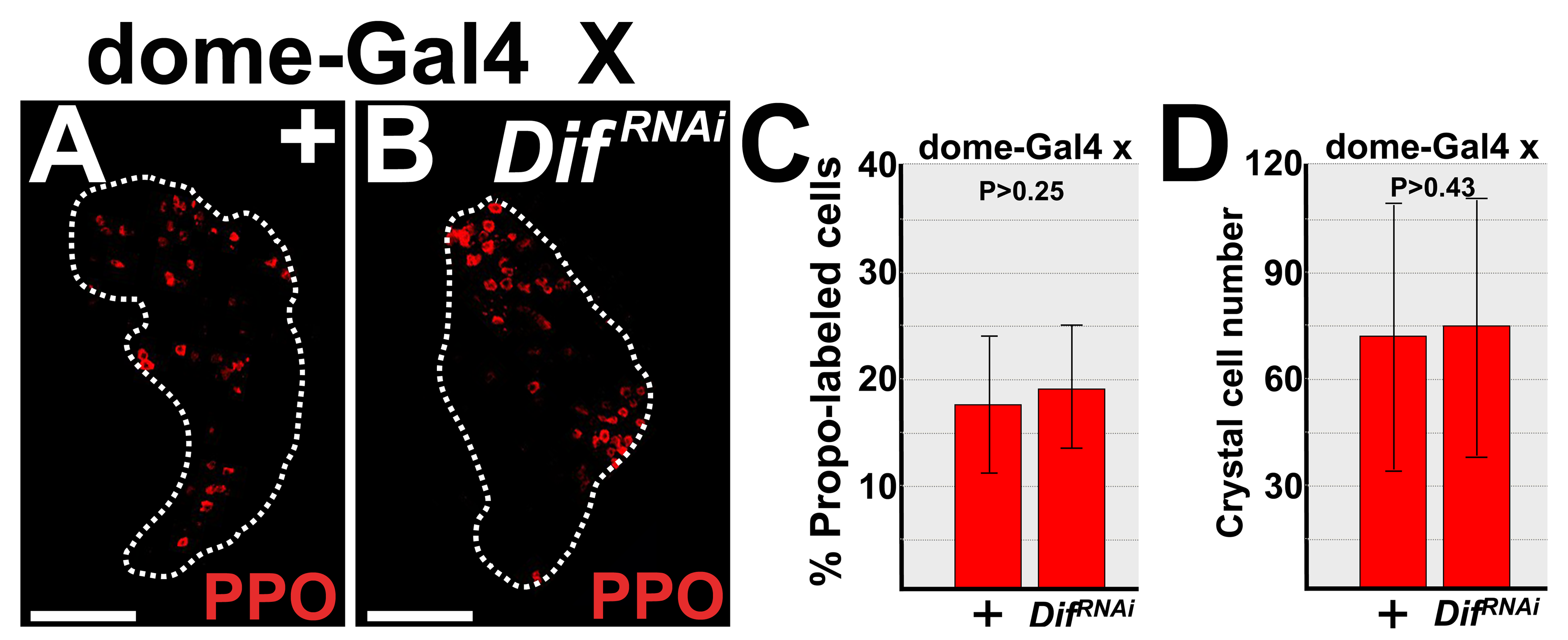

Supplement: S5 Fig — (A,B) Crystal cell numbers were unchanged in the lymph glands from dome-Gal4 driven UAS-DdlRNAi (DifRNAi) larvae compared to controls. Lymph glands from mid-third instar larvae. White dotted lines delineate the entire lymph gland. Scale bars: 50 μm. (C) Histogram showing the percentage of crystal cells in controls and DifRNAi lymph glands. (D) Histogram showing the number of crystal cells in controls and DifRNAi lymph glands. (C,D) Student’s t-test; error bars show standard deviation; control and DifRNAi (n = 17); P value is as shown. (TIF) [file pone.0155372.s005.tif]

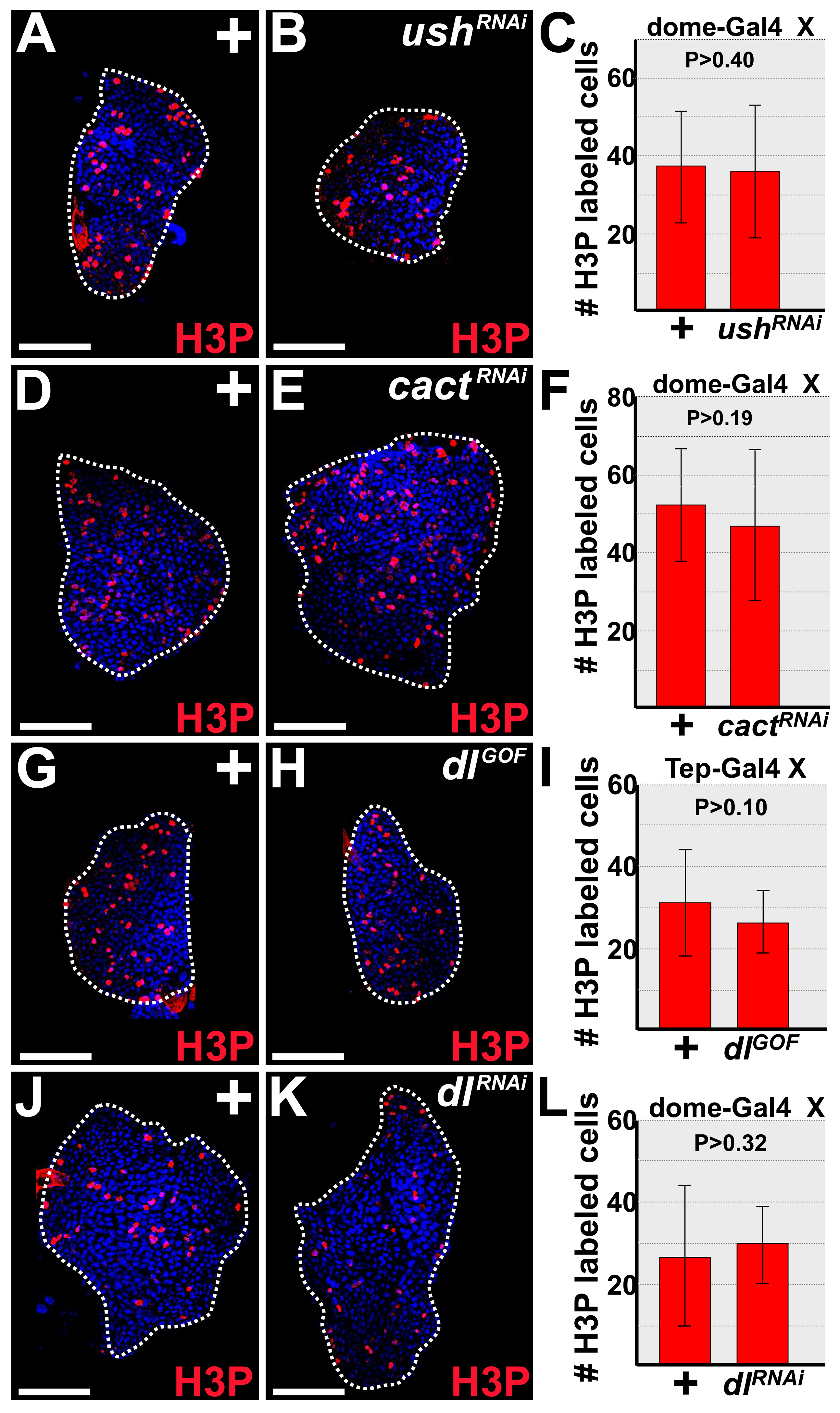

Supplement: S6 Fig — dome-Gal4 females were crossed to control (+) males or males that carry UAS-ushRNAi, UAS-cactRNAi, UAS-dl or UAS-dlRNAi transgenes. (A-C) Knockdown of Ush (ushRNAi), (D-F) knockdown of Cact (cactRNAi), (G-I) over-expression of Dorsal (dlGOF) or (J-L) knockdown of Dorsal (dlRNAi) had no effect on proliferation as measured with phosphohistone H3 (H3P) antibody staining. Histograms showing (C,F,I,L) the number of H3P labeled cells was not significantly different in lymph glands in which the expression of ush, cact or dl was altered in prohemocytes compared to their respective controls. Student’s t-test; error bars show standard deviation; P value is as shown; control and ushRNAi (n = 24); control and cactRNAi (n = 21); control and dlGOF (n = 19); control and dlRNAi (n = 15). Lymph glands from mid-third instar larvae. White dotted lines delineate the entire lymph gland. Scale bars: 50 μm. (TIF) [file pone.0155372.s006.tif]
